# A Simple and Efficient Non‐Noble Cathode Catalyst Based on Carbon Hollow Nanocapsules Containing Cobalt‐Based Materials for Anion Exchange Membrane Water Electrolyzer

**DOI:** 10.1002/smll.202411019

**Published:** 2025-02-03

**Authors:** Sivaprakasam Radhakrishnan, Shanmugam Ramakrishnan, Santhosh Kumar Jayaraj, Mohamed Mamlouk, Byoung‐Suhk Kim

**Affiliations:** ^1^ Department of Chemistry School of Sciences and Humanities SR University Warangal Telangana 506371 India; ^2^ School of Engineering Newcastle University Tyne and Wear UK; ^3^ Department of Organic Materials & Textile Engineering Jeonbuk National University 567 Baekje‐daero Jeollabuk‐do 54896 Republic of Korea; ^4^ Department of JBNU‐KIST Industry‐Academia Convergence Research Jeonbuk National University 567 Baekje‐daero, Deokjin‐gu, Jeonju‐si Jeollabuk‐do 54896 Republic of Korea

**Keywords:** electrocatalyst, hollow structure, metal–organic frameworks, water electrolyzer

## Abstract

An efficient approach for fabrication of non‐noble metal‐based electrocatalyst is desirable for designing the energy storage and conversion devices in real‐world usages due to low cost and excellent catalytic properties. The preparation of hollow carbon capsules (HCC) containing cobalt (Co)‐based electrocatalyst is reported by a simple synthesis process without using templates for the first time. Initially, cobalt phenylphosphonate (Co‐MOF) nanorods are fabricated through a simple hydrothermal approach. The as‐formed Co‐MOF is covered with a thin coating of polydopamine (DP‐Co‐MOF) through chemical polymerization of dopamine in Tris‐HCl (pH 8.5). The DP‐Co‐MOF is used as self‐degraded template for the formation of HCC under pyrolysis. The formation mechanism and hydrogen evolution reaction (HER) activity of HCC are investigated. The hollow structure derived under N_2_ exhibits a low overpotential (295 mV at 100 mA cm^−2^) with excellent stability (90.98%) for 150 h, which is further verified by density functional theory (DFT) calculations. Finally, the designed anion exchange membrane (AEM) water electrolyzer based on C─Co─N as cathode delivers a current density of 500 mA cm^−2^ (at 2.19 V) and 1000 mAcm^−2^ (at 2.33 V) in 1.0 m KOH at 60 °C. The fabricated new Co‐based electrocatalyst is highly beneficial for the fabrication of cost‐effective and high‐performance AEMWEs.

## Graphical abstract

This study demonstrates the new preparation of hollow carbon capsules as cathode materials for demonstrating the anion exchange membrane (AEM) water electrolyzer applications. The fabricated AEM water electrolyzer device delivers excellent analytical performance with high current density and long‐term stability.

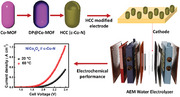

## 1 Introduction

Renewable energy‐related research activities are growing rapidly in different directions due to fast depletion of fossil fuels and associated environmental issues.^[^ ^1^, ^2^, ^3^, ^4^ ^]^ Hence, the inventions are focused on the development of renewable energy though sustainable sources that are affordable, safe, and clean.^[^ ^5^ ^]^ Hydrogen has outstanding potential as a future fuel compared to non‐conventional sources like wind, and solar due to its merits such as zero carbon emissions, and abundant inexpensive sources (e.g., water). However, ≈80% of the world's hydrogen is produced by conventional methods that have several disadvantages including low purity, and the production of greenhouse gases.^[^ ^6^, ^7^ ^]^ Alternatively, the electrical generation of hydrogen from water is the most efficient and safe method to produce quality hydrogen.^[^ ^8^, ^9^ ^]^ However, the electrochemical water oxidation reaction requires high performance electrocatalysts (including those involving Pt, RuO_2_, and IrO_2_) due to its sluggish reaction kinetics.^[^ ^10^ ^]^ Although these noble metal catalysts deliver good electrochemical performance in water‐electrolysis, the major problems associated with large‐scale commercialization of such devices are their high‐cost, and scarcity.^[^ ^11^ ^]^ Currently, researchers are following two strategies to overcome these issues i) development of high‐efficiency electrocatalyst based on transition metal materials^[^ ^12^, ^13^, ^14^ ^]^ and ii) addition of trace amount of noble metals through preparation of alloys with transition metals or deposition with carbon or metal substrates without affecting the water splitting efficiency.^[^ ^15^, ^16^, ^17^ ^]^

In the past several years, significant developments have been demonstrated based on transition metals, and carbon materials and their modified derivatives as alternatives to conventional expensive catalysts.^[^ ^12^, ^13^, ^14^, ^15^, ^16^, ^17^, ^18^, ^19^, ^20^, ^21^ ^]^ Notably, the well‐known non‐noble cobalt‐based metal catalysts (metallic Co, Co_3_N, Co_4_N, CoP, Co_2_P, CoS_2_, CoSe_2_, Co_2_P_2_O_7_, etc.) are attracting much attention in water electrolysis applications.^[^ ^22^, ^23^, ^24^, ^25^, ^26^, ^27^, ^28^, ^29^, ^30^ ^]^ In particular, hollow structured cobalt compounds are highly promising electrode active materials for water splitting applications due to their unique cavity structure, large surface area, rich active sites, good stability, excellent catalytic activity, low toxicity, and the various beneficial effects of a hollow structure.^[^ ^25^, ^26^, ^27^, ^28^ ^]^ Furthermore, the fabrication of hollow structures with a protective conducting layer over the cobalt catalyst is much more beneficial in reducing the dissolution of cobalt active sites in acidic or alkaline conditions and in preventing catalytic particle agglomeration and relocation.^[^ ^25^, ^26^, ^27^, ^28^ ^]^ Hollow structure‐based materials were achieved using various approaches including template‐based methods (hard and soft), Ostwald ripening, the Kirkendall effect, and galvanic replacement.^[^ ^31^ ^]^ Conventional template‐based methods have several disadvantages such as time‐consuming, tedious processes, involving corrosive chemicals, and low product‐yield.^[^ ^32^ ^]^ Alternatively, self‐template‐based methods are very attractive and have received much interest in recent years due to their benefits including scalable production, highly reproducible process, and use of a single feedstock.^[^ ^33^ ^]^ However, a simple, and highly reproducible approach for the fabrication of hollow‐structure‐based nanostructures remains a challenge.^[^ ^33^, ^34^, ^35^, ^36^ ^]^

In this study, we successfully prepared Co‐based electrocatalyst (Co_2_P_2_O_7_ and Co_2_P) containing hollow carbon capsules (HCC; C─Co─N) through a simple approach without surfactants, etching and or hazardous chemicals. Surprisingly, the hollow carbon capsule (C─Co─N) electrocatalyst demonstrated a good electrochemical performance in a hydrogen evolution reaction (HER) compared to other controlled cobalt‐based electrocatalyst. The C─Co─N electrocatalyst delivered a low overpotential of 295 mV at 100 mA cm^−2^ with excellent stability. The structural stability and hydrogen interactions of the C─Co─N were studied in detail by density functional theory (DFT). To confirm the real applicability of the developed HER catalyst, we designed an AEM water electrolyzer using a C─Co─N electrocatalyst as a cathode catalyst and studied the device characteristics in 0.1 and 1.0 m KOH at 20 and 60 °C. This newly developed self‐template assisted preparation of hollow carbon nanocapsules could open a new avenue for designing and synthesizing high‐performance non‐noble transition metal‐based electrocatalysts for alkaline AEM water electrolyzers for hydrogen generation applications.

## 2 Results and Discussion

### 2.1 Preparation and Investigation of Hollow Carbon Capsules (HCC)

**Figure 1:**
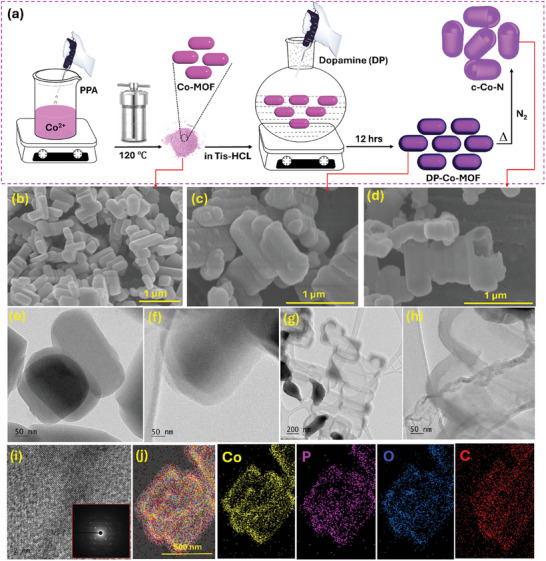
a) Schematic diagram for the formation of hollow carbon capsules (HCC) containing Co‐based electrocatalyst (C─Co─N). FE‐SEM images of b) Co‐MOF; c) DP‐Co‐MOF; d) C─Co─N electrocatalyst. HR‐TEM images of e) Co‐MOF; f) DP‐Co‐MOF; g–i) C─Co─N and j) Energy dispersive X‐ray spectroscopy mapping of C─Co─N electrocatalyst.

The preparation of hollow carbon capsules (HCC) containing Co‐based electrocatalyst is schematically represented in **Figure** **1**a. We followed a simple two‐step synthesis procedure to produce HCC without aid templates/surfactants or etching hazardous chemicals. Initially, the physiochemical properties of the Co‐MOF prepared using a simple hydrothermal method via mixing the Co precursor (as a metal source) with phenylphosphonic acid (as an organic linker) in a water and ethanol mixture were studied by various analytical characterizations. Figures 1b and S2a (Supporting Information) show the FE‐SEM images of the as‐obtained Co‐MOF nanostructure. Highly uniform 1D small nanorod morphologies with a smooth surface were observed. The average thickness of the Co‐MOF nanorods was 205 ± 20 nm. EDS mapping and spectra were obtained for the Co‐MOF product as depicted in Figure S3 (Supporting Information). As expected, all elements such as C, Co, P, and O showed in the Co‐MOF nanorod morphologies (Figure S4, Supporting Information) with a uniform distribution throughout the 1D nanorod. In addition, the interior morphologies of as‐prepared Co‐MOF were further studied using TEM (Figures 1e and S2b, Supporting Information). Perfect 1‐D nanorod morphologies with an average thickness of 205 ± 10 nm were observed, in good agreement with FESEM observations (Figure 1b). The phase purity of the as‐derived Co‐MOF was studied by XRD (Figure S5, Supporting Information). The derived peak patterns were aligned with the orthorhombic crystal shape of cobalt phenylphosphonate (PDF#51‐2096), confirming the purity of the as‐obtained Co‐MOF.^[^ ^37^ ^]^ It is well‐known that controlled preparation of metal phosphonate nanostructures is a complicated process because the metal phosphonate co‐ordination reaction is kinetically very fast, resulting in the formation of uncontrolled precipitation through the reaction between the metals and phosphonic acid linker.^[^ ^37^, ^38^ ^]^ Here, we simply achieved highly uniform 1D nanorods without any templates using a water and ethanol mixture under a facile hydrothermal approach. The key advantages of the present Co‐MOF preparation are that it is simple, highly reproducible, and scalable. The large‐scale preparation with reproducible morphologies was confirmed through increasing the concentration of precursors under similar conditions. Subsequently, the as‐obtained Co‐MOF 1D nanorods were used as precursors for the preparation of polydopamine‐coated Co‐MOF (DP‐Co‐MOF; Figure S1bSupporting Information) through self‐polymerization of dopamine over the Co‐MOF in Tris‐HCl (pH 8.5).^[^ ^39^ ^]^ The surface morphology of the DP‐Co‐MOF product was verified by FESEM (Figures 1c and S2c, Supporting Information) and TEM (Figures 1f and S2d, Supporting Information). It clearly shows uniform coating of polydopamine on top of the entire Co‐MOF 1D nanorod surface and increased surface roughness compared to bare Co‐MOF. The estimated thickness of the polydopamine coating layer over the Co‐MOF nanorod was 50 ± 4 nm (Figure 1f). EDS mapping was performed for the DP‐Co‐MOF product and demonstrated the existence of all elements with a uniform distribution (Figure S6, Supporting Information). XRD was conducted for the as‐obtained DP‐Co‐MOF product to identify any alteration in the crystal structure of the Co‐MOF after polydopamine coating. Figure S7 (Supporting Information) shows the XRD spectrum of the DP‐Co‐MOF product, where the observed peak patterns were identical to those of the Co‐MOF crystal structure, suggesting no effect of the polydopamine coating on the crystal structure of Co‐MOF due to the amorphous characteristics of polydopamine. Subsequently, the DP‐Co‐MOF was subjected to thermal treatments in N_2_ and air atmospheres to synthesize Co‐based nanomaterials. For example, the DP‐Co‐MOF was pyrolyzed at 800 °C under a N_2_ atmosphere (C─Co─N) and the obtained black residues (Figure S1d, Supporting Information) were studied by various techniques. Figures 1d and S2e (Supporting Information) showed the FESEM images of the C─Co─N electrocatalyst and uniform 1D nanorods with a morphology of Co‐MOF. The surface roughness greatly decreased compared to that of DP‐Co‐MOF (Figure 1c) due to the complete conversion of polydopamine into carbon shells at elevated temperature. Interestingly, the C─Co─N electrocatalyst appeared as a hollow structure containing several cavities in the carbon shell (Figure 1d). FESEM mapping studies showed the presence of all elements over the nanorod morphologies with uniform distribution (Figure S8, Supporting Information). Interior morphologies and hollow structured properties of the C─Co─N electrocatalyst were verified by TEM. Figures 1g and S2f (Supporting Information) show TEM images of the C─Co─N electrocatalyst, indicating a clear 1D hollow nanostructure. The estimated average thickness of the C─Co─N hollow structure was 230 ± 10 nm and the thickness of the carbon shell (derived from polydopamine coating) was 43 ± 5 nm (Figure 1h). The well‐defined lattice fringes from HR‐TEM (Figure 1i) and bright dotted lines from the SAED pattern of C─Co─N (Inset of Figure 1i) confirmed the polycrystalline nature of the as‐derived C─Co─N product from DP‐Co‐MOF under pyrolysis in N_2_. TEM EDS mapping studies were carried out for the C─Co─N product as depicted in Figure 1j. All the expected elements (Co, P, C, and O) existed in the hollow structure, and the Co‐based products were observed at the wall of the hollow product as hollow carbon capsules (HCC).

**Figure 2:**
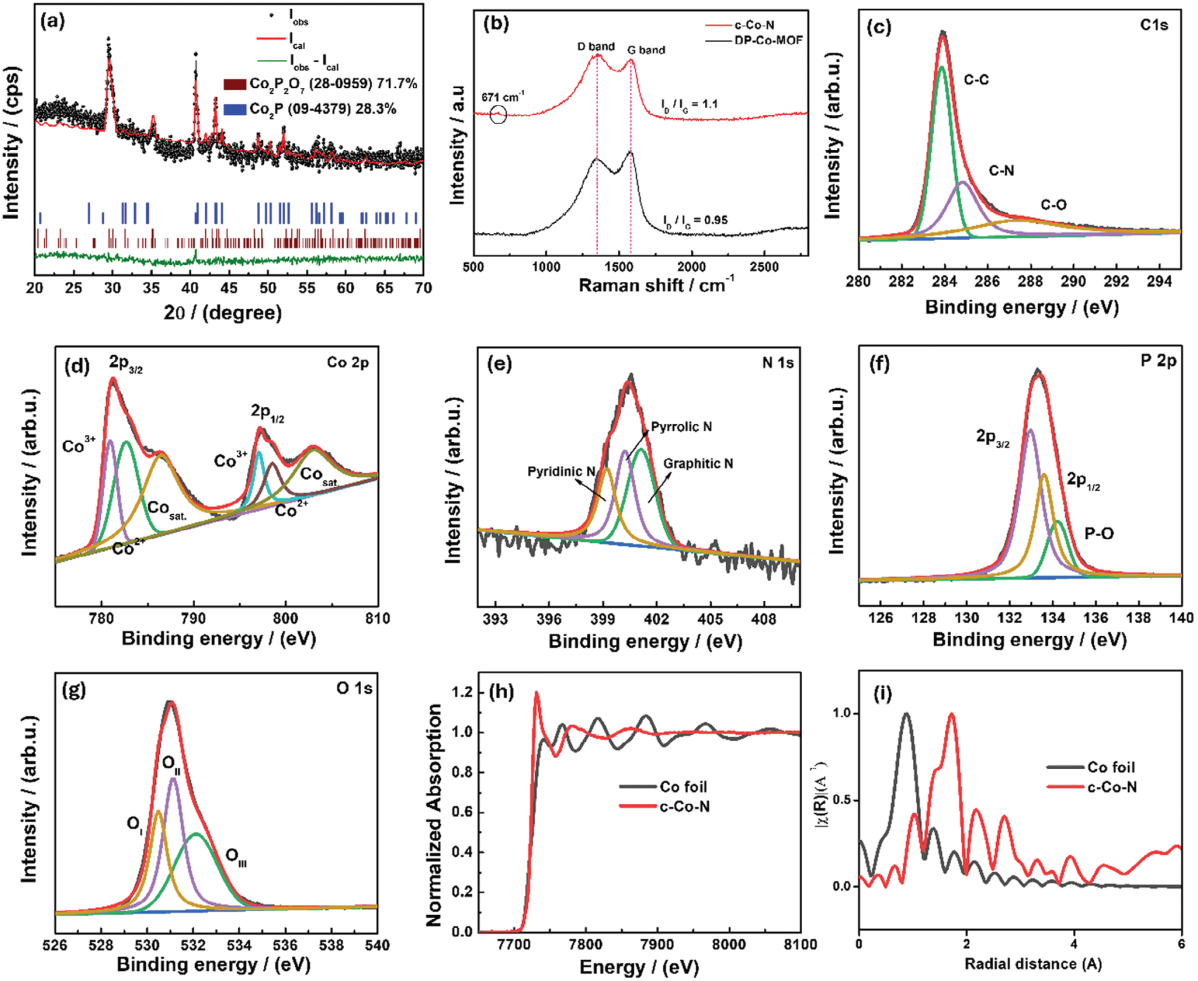
a) Rietveld fits of the XRD data of C─Co─N product; (g‐l) XPS spectrum of C─Co─N electrocatalyst; (g); b) Raman spectra of DP‐Co‐MOF and C─Co─N electrocatalyst c–g) XPS high‐resolution spectrum C─Co─N electrocatalyst; c) C, d) Co, e) N, f) P, and g) O; h, i); XAS analysis of C─Co─N and Co‐foil, i) Co─K edge XANES spectra of Co foil and C─Co─N.

The crystal structure and phase formation of the as‐obtained Co‐based HCC (C─Co─N) were verified by XRD, as shown in Figure S9 (Supporting Information). The obtained peak pattern of the C─Co─N electrocatalyst perfectly matched the crystal structures of Co_2_P_2_O_7_ (ICSD 280 959) and Co_2_P (ICSD 94 379). Further, the XRD Rietveld fit with the as‐obtained XRD pattern of the C─Co─N verified the major contribution of the crystal structure (**Figure** **2**a). Rietveld fit studies confirmed the major contribution of Co_2_P_2_O_7_ (71.7%) over the Co_2_P (28.3%) crystal structure in the as‐derived C─Co─N electrocatalyst, obtained from the pyrolysis of DP‐Co‐MOF in N_2_. The XRD studies clearly demonstrated the formation of a mixed‐phase C─Co─N electrocatalyst. In contrast, when the same DP‐Co‐MOF was treated in an air atmosphere under similar conditions, and the obtained grey product (C─Co─A; Figure S1f, Supporting Information) was characterized by XRD, as shown in Figure S9 (Supporting Information). The obtained XRD peak patterns perfectly matched the monoclinic crystal structure of Co_2_P_2_O_7_ (ICSD 280 959; space group of P 1 21/c 1) with no deviations or additional peaks. These results confirmed the formation of pure Co_2_P_2_O_7_ crystal structure through thermal treatment of DP‐Co‐MOF in an air atmosphere (C─Co─A). However, the surface morphologies of the C─Co─A product (Figure S10, Supporting Information) were different from those of DP‐Co‐MOF (Figure 1c) and C─Co─N (Figure 1d). Figure S10 (Supporting Information) shows the FESEM images of the C─Co─A product, which contained aggregated small, spherical particles due to complete decomposition of organic functional groups in the DP‐Co‐MOF product at elevated temperature in air. Further, EDS mapping studies implied the presence of cobalt, phosphorus, and oxygen in the C─Co─A product with uniform distribution over the particle‐like morphologies (Figure S11, Supporting Information). As is evident from the EDS spectrum (Figure S12, Supporting Information), the intensities of carbon peaks were greatly suppressed due to the rapid decomposition of carbon at high temperature in an air and the presence of pure cobalt pyrophosphate with particle‐like morphology.

For comparison, we conducted thermal treatment in both air (Co─A; Figure S1e, Supporting Information) and N_2_ (Co‐N; Figure S1c, Supporting Information) atmospheres for the as‐prepared Co‐MOF product under similar conditions. Figure S13 (Supporting Information) shows the FESEM images of the Co‐MOF product calcinated under N_2_ (Co─N) and the observed porous 1D nanorod morphologies. The deep morphologies of the Co─N product were further observed by TEM as depicted in Figure S14 (Supporting Information). The porous 1D nanorods were formed by assembling small nanoparticles (≈11 nm), showing characteristic features of MOF‐based products under thermal treatment in inert conditions. The retention of 1‐D structures after thermal treatment of Co‐MOF is due to the complete conversion of organic‐linker (phenylphosphonic acid) in the Co‐MOF into carbon. To verify the phase purity of the as‐derived Co─N product, XRD measurements were performed as depicted in Figure S9 (Supporting Information). The observed peak patterns exactly matched the crystal structures of Co_2_P_2_O_7_ (ICSD 280 959) and Co_2_P (ICSD 94 379) and confirmed the formation of mixed‐phase Co‐based products from Co‐MOF. In addition, the same Co‐MOF treated in air condition (Co─A; Figure S1e, Supporting Information), formed a surface morphology of irregular particle‐like structures (Figure S15, Supporting Information), different from the morphology of the Co─N product (Figure S13, Supporting Information). This irregular particle morphology is due to the complete elimination of organic linkers at elevated temperature in air. The EDS mapping (Figure S16, Supporting Information) and spectra (Figure S17, Supporting Information) clearly confirmed the fine distribution of Co, P, and O within the particle morphologies. The crystal structure of the as‐obtained Co─A product was confirmed by XRD (Figure S9, Supporting Information) and the observed peak pattern completely matched that of the Co_2_P_2_ O_7_ (ICSD 280 959) crystal structure, with no additional peaks or impurities. Raman spectroscopy was applied to derive information on the nature of carbon, graphitization degree, and defect. Figure 2b displays comparative Raman spectra of DP‐Co‐MOF and c‐Co─N electrocatalyst. As expected, two distinguished peaks were present for both cobalt products, which correlated to the disordered carbon (D band) and ordered carbon (G band). This result confirmed the presence of carbon in DP‐Co‐MOF and C─Co─N electrocatalyst. The enhancement of the D band arose due to the vibration of sp^3^ hybridized carbon in disordered graphitic planes. Similarly, the G band enhancement appeared through E_2g_ vibration of sp^2^ hybridized carbon in the ordered graphitic carbon (degree of graphitization).^[^ ^40^ ^]^ Here, the G band was greatly suppressed for C─Co─N electrocatalyst compared to DP‐Co‐MOF material. This indicates larger amount of disordered carbon in the C─Co─N electrocatalyst after thermal treatment of DP‐Co‐MOF in N_2_. Further, the degree of disorder (defect) in carbon material was confirmed by measuring the I_D_/I_G_ value, which was estimated to be 1.1 for the C─Co─N electrocatalyst, which was significantly higher than that of DP‐Co‐MOF (0.95). The higher I_D_/I_G_ indicates the presence of greater numbers of defects and disorders in the as‐derived C─Co─N electrocatalyst, which is highly beneficial for access of large electrolyte ions into the material modified surface to modify the surface and obtain improved electrocatalytic performance. Further, a minor peak was noticed at 671 cm^−1^, which was correlated to the P─O─P bridge in the cobalt (II) pyrophosphate.^[^ ^41^ ^]^ Raman spectroscopy confirmed the effective formation of highly defective carbon product and the presence of Co‐product in the as‐derived C─Co─N hollow structure from DP‐Co‐MOF after pyrolysis. Further, nitrogen adsorption–desorption isotherm measurements were conducted for the Co‐MOF, C─Co─N, and Co─N products to verify their surface areas (Figure S18, Supporting Information). The C─Co─N product exhibited a high surface area (75.86 m^2^g^−1^) compared to Co‐MOF (14.30 m^2^g^−1^) and Co─N (21.98 m^2^g^−1^) products. The estimated pore size was higher for the C─Co─N (24.7 nm) derived from the Barett–Joyner–Halenda model and was significantly higher than those of the Co‐MOF (14.8 nm) and Co─N (11.3 nm) products. The stronger mesoporous characteristics of the C─Co─N electrocatalyst have several attractive benefits including rapid access of the inner part of the cobalt product to electrolyte ions for enhancing catalytic efficiency of the electrode. To further verify the chemical composition and oxidation state of the as‐derived C─Co─N electrocatalyst, we used X‐ray photoelectron spectroscopy (XPS; Figures 2c–g and S19, Supporting Information). The as‐received XPS survey spectrum and high‐resolution spectra of the C─Co─N electrocatalyst are displayed in Figure S19 (Supporting Information). As expected, all the major elements of cobalt, phosphorus, nitrogen, carbon, and oxygen were present in the survey spectrum of C─Co─N. Further, the oxidation states of the elements in the C─Co─N electrocatalyst were verified through the high‐resolution spectrum of each element, and that of Co 2p was displayed in Figure 2d. The high‐resolution spectrum of Co 2p showed the difference peaks in the binding energy between 780–808 eV. The peaks observed at 781.2 and 797.5 eV were correlated to Co 2p_3/2_ and Co 2p_1/2_, respectively, with satellite peaks (786 and 802.9 eV) showing the presence of Co^3+^ and Co^2+^ in the C─Co─N electrocatalyst.^[^ ^28^ ^]^ The peak at 133.3 eV is linked to the P 2p energy levels of the C─Co─N electrocatalyst (Figure 2f). The P 2p_3/2_ and P 2p_1/2_ peaks were observed at 133.2 and 133.9 eV, respectively. Further, the peak observed at 134.4 eV is due to the bonding characteristics of phosphorus with a carbon lattice structure.^[^ ^42^ ^]^ The peak at 133.6 eV was attributed to the presence of chemically bonded P‐O atoms. Figure 2c shows the C 1s high‐resolution spectrum of the C─Co─N product, in which three distinguished peak positions at 284, 284.5, and 286 eV are related to C═C from aromatic, aliphatic, and graphitic bonds, C─C and C─N bonds, respectively.^[^ ^43^ ^]^

X‐ray absorption spectroscopy (XAS) was used to further understand the accurate surface coordination states of cobalt in the C─Co─N electrocatalyst (Figure 2h,i). Figure 2h shows the XAS near‐edge (XANES) energy position of the C─Co─N electrocatalyst and Co‐foil (as a reference). In these XANES data, the intensity at 7736 eV for C─Co─N was improved significantly compared to the reference Co foil. This enhanced intensity of C─Co─N electrocatalyst due to the fact that the changes of local electronic density and coordination environment of cobalt through bonding with oxygen and phosphorus atoms. Figure 2i depicts the Fourier‐transformed X‐ray absorption fine structure (EXAFS) spectra of C─Co─N and Co‐foil. In the EXAFS spectrum of C─Co─N, the intensity of peak position at 1 – 1.80 Å was improved than the Co foil due to the interaction of Co with other atoms (Co‐O and Co‐P).^[^ ^44^, ^45^ ^]^

### 2.2 Electrocatalytic Behavior of Hollow Carbon Capsules (HCC)

**Figure 3:**
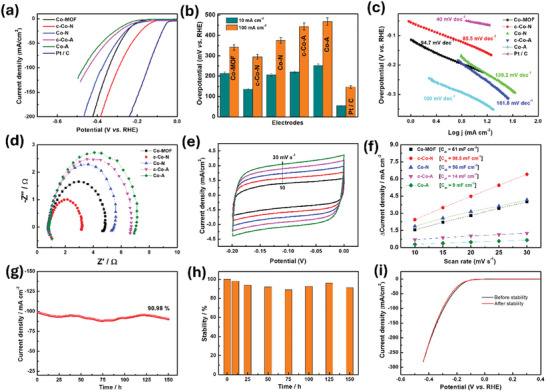
a) HER LSV profiles of different cobalt electrocatalyst in 1.0 m KOH at scan rate of 1 mV s^−1^(iR‐corrected). b) Bar graph of overpotential against different Co‐based electrocatalyst, c) Tafel plots for different Co‐based electrocatalyst. d) Electrochemical impedance spectroscopy of different electrodes, e) CVs of C─Co─N electrocatalyst at different scan rates from 10–30 mVs^−1^. f) corresponding linear plot of change in current density versus scan rate. g) amperometric stability of the cCo‐N electrocatalyst, h) Bar graph of stability against time, and i) Comparative LSV curves before and after stability measurement.

The hydrogen evolution reaction (HER) electrocatalytic performance of as‐derived various Co‐based electrocatalyst was studied under the alkaline condition (1 m KOH) and compared with that of commercial Pt/C (20%) catalyst and Co‐MOF material. **Figure** **3**a shows the linear sweep voltammogram (LSV) curves of Co‐based electrocatalyst recorded at a sweeping scan rate of 1 mV s^−1^ and displayed after *iR* correction. The LSV behaviors clearly show HER activity for all the all materials including Co‐MOF, Co─A, Co‐N, C─Co─N, and C─Co─A (Figure 3a). Improved HER performance was observed for the C─Co─N electrocatalyst compared to other cobalt‐based materials. In particular, the C─Co─N electrode reached low overpotentials of 137 and 295 mV to achieve current densities of 10 (η_10_) and 100 mA cm^−2^ (η_100_), respectively (Figure 3b). The obtained overpotential values were lower than those of the other cobalt product‐ modified electrodes of Co‐MOF (206 mV @ η_10_; 342 mV @ η_100_), Co‐N (214 mV @ η_10_; 375 mV @ η_100_), C─Co─A (221 mV @ η_10_; 444 mV @ η_100_), and Co─A (252 mV @ η_10_; 468 mV @ η_100_) (Figure 3b). The enhanced HER activity of as‐derived C─Co─N electrocatalyst is due to the unique advantages of good catalytic activity of Co─P with a cavity structure, which provides various attractive characteristics such as ensemble effects during the HER process, nano confinement effects, good permeability, large surface area, good adsorption, diffusion of electrolyte ions, and enhanced catalytic active sites.^[^ ^46^, ^47^ ^]^ Further, the nitrogen‐doped carbon shell enhances the electrical conductivity, catalytic activity, and desorption of catalytic products from the catalyst surface.^[^ ^15^, ^41^, ^43^, ^45^ ^]^ The HER kinetics of the prepared electrocatalysts was assessed by calculating the Tafel slopes from equation, η = a + b* logǀ*j*ǀ, where η, a, b and j are the η_10_ (mV), intercept, Tafel slope, and absolute rate of current density, respectively.^[^ ^48^ ^]^ The estimated Tafel slope values for prepared electrocatalysts are presented in Figure 3c. Among the various Co‐based electrocatalyst, the C─Co─N delivered a low Tafel slope (85.5 mV dec^−1^) compared to Co‐MOF (94.7 mV dec^−1^), Co‐N (139.2 mV dec^−1^), C─Co─A (161.8 mV dec^−1^), and Co─A (100 mV dec^−1^). This clearly suggests that the hollow carbon capsules containing cobalt‐based nanomaterials were highly electroactive with enhanced charge transfer kinetics toward the HER in alkaline conditions. According to the literature reports, based on the Tafel slope there are three different rate determining, as represented by the following equations.^[^ ^49^ ^]^ Initially, hydrogen is electrochemically adsorbed onto the electrocatalyst surface (Catalyst‐H_ad_), while OH^−^ ions are generated as water molecules dissociate during HER. Finally, H₂ is produced from Catalyst – H_ad_ via two distinct pathways: the Heyrovsky and Tafel steps. Based on the Tafel slope, the formation of H₂ gas from water reduction over the C─Co─N electrocatalyst occurs through the Volmer‐Heyrovsky step.

(1) $$\begin{array}{ccc} H_{2}O+\mathrm{Catalyst}+e^{-} & & \to \mathrm{Catalyst}\\ & & \;\;\;\;-\;\;H_{\mathrm{ad}}+OH^{-}\\ & & \;\;\;\;\left(\mathrm{Volmer}\;\mathrm{step};120\;\mathrm{mV}\;\;{\mathrm{dec}}^{-1}\right) \end{array}$$

(2) $$\begin{array}{ccc} H_{2}O+\mathrm{Catalyst}-H_{\mathrm{ad}}+e^{-} & & \to H_{2}+\mathrm{Catalyst}+OH^{-}\\ & & \;\;\;\;\left(\text{Heyrovsky step; 40 mV}\;\;{\mathrm{dec}}^{-1}\right) \end{array}$$

(3) $$2\mathrm{Catalyst}-H_{\mathrm{ad}}\to H_{2}+2\mathrm{Catalyst}\left(\text{Tafel step; 30 mV}\;\;{\mathrm{dec}}^{-1}\right)$$

Subsequently, the electrical properties of the modified electrodes were assessed by electrochemical impedance spectroscopy (EIS) studies as depicted in Figure 3d. The recorded EIS results are presented as Nyquist curves, and the derived charge‐transfer resistance (R_CT_) was significantly altered for each modified electrocatalyst surface with Ni foam. The lowest R_CT_ (2.2 Ω) was that of the C─Co─N electrocatalyst compared to the other prepared electrocatalyst: Co‐MOF (3.82 Ω), Co─N (4.46 Ω), C─Co─A (5.58 Ω), and Co─A (5.87 Ω). The number of catalytic active sites on the modified electrodes directly depends on the electrochemically active surface area (ECSA), which calculated from *C*_dl_ values by measuring the cyclic voltammetry in the non‐Faradaic region. We recorded CVs for the modified electrodes at various scan rates from 10 to 30 mV s^−1^ (Figure 3e). Based on CV measurements, the linear plot showed the differences in current density against anodic and cathodic current profile (measured at −0.1 V) and scan rates for all the cobalt electrocatalyst. The derived slope of the linear plot is twice the value of *C*_dl_, and the obtained *C*_dl_ values are provided in the inset of Figure 3f. The maximum *C*_dl_ was obtained for C─Co─N (98.5 mF cm^−2^) electrocatalyst compared to *C*_dl_ values of Co‐MOF (61 mF cm^−2^), Co‐N (56 mF cm^−2^), C─Co─A (14 mF cm^−2^), and Co─A (9 mF cm^−2^) electrocatalyst. The highest *C*_dl_ of the C─Co─N electrocatalyst demonstrated the presence of rich catalytic active sites in HCC material and a high surface area with a good conducting layer of nitrogen‐doped carbon over the HCC material, which provided improved HER with good stability in alkaline conditions. The obtained *C*_dl_ were perfectly corroborated by the overpotential (η) values of the Co‐based materials. Electrocatalytic performance was higher for the HCC (C─Co─N) modified electrode toward HER compared to Co‐MOF, C─Co─A, Co─N, and Co─A.

Further, the analytical HER performances (such as low over potential and Tafel slope and high stability) of HER at HCC electrodes were superior to many and comparable to those of a few cobalt‐based electrocatalyst reported in recent literature including Co_2_P_2_O_7_, CoPS_3_, Co_2_P/CoP, Co_9_S_8_, Co‐Mo‐P, Co_3_S_4_‐MoS_2_, NiCoSe, and Ni‐CoP/Co_2_P (Table S2, Supporting Information). The stability of a catalyst is important for real‐world energy harvesting applications. Here, we verified the stability of our prepared C─Co─N electrocatalyst by chronoamperometry (CA) and linear sweep voltammetry (LSV) techniques. Figure 3g shows the continuous CA study at an applied potential corresponding to 100 mA cm^−2^ for 150 h using a C─Co─N electrocatalyst with stabilities of 98.20%, 94.15%, 92.00%, 89.48%, 92.70%, 96.00%, and 90.98% for 10, 25, 50, 75, 100, 125, and 150 h, respectively (Figure 3h). A ≈90.0% current density was retained at end of the CA stability test compared to the initial value. Figure 3i shows the comparative LSV profiles of the C─Co─N electrocatalyst before and after 150 h of CA stability measurement. A slight drift in the LSV profile was noticed, suggesting excellent stability of the present modified electrode. That stability was much better than many reports of Co‐based electrocatalyst (Table S2, Supporting Information).

The characterization of prepared C─Co─N electrocatalyst after stability measurement is very important to validate the durability of the catalyst for real‐time applications. Thus, we analyzed the materials for chemical composition, and morphology. Measurement after stability studies were conducted using various techniques such as FESEM, EDS‐mapping and XPS measurements. Figure S20a (Supporting Information) shows the FESEM surface morphologies of the C─Co─N electrocatalyst after the durability studies. The nanorod morphologies of C─Co─N electrocatalyst was changed during the durability studies due to the continuous evolution of hydrogen gas.^[^ ^47^ ^]^ Elemental mapping was conducted for the C─Co─N electrocatalyst after the stability measurement (Figure S20b–f, Supporting Information). All expected elements were present and distributed uniformly over the morphology of HCC, suggesting the as‐fabricated HCC demonstrated a good stability toward HER electrocatalytic applications for long‐term usages. XPS was performed as depicted in Figure S20g–i (Supporting Information) to verify the changes of elemental composition and electronic configurations of C─Co─N electrocatalyst after the stability studies. The elements present before and after stability were identical with a slight shift in the binding energies and peak intensities, suggesting good stability of C─Co─N electrocatalyst for a longer cycle‐life. The comparative XPS parameters of the C─Co─N electrocatalyst before and after the stability studies are provided in Table S1 (Supporting Information). As in Table S1 (Supporting Information), the phosphorus quantity significantly decreased from 11.11% to 3.76%, whereas other elements decreased only slightly. However, the atomic% Co content was increased after the stability measurement (14.46%) compared to that before the stability studies (7.21%). Similarly, oxygen content increased from 35.22% to 37.03% due to the longer electro‐catalytic reaction of C─Co─N electrocatalyst under alkaline conditions. The post‐HER analysis studies implied that the fabricated electrode retained the electronic structure with slight changes of the elemental composition, which did not much significantly affect the electrocatalytic performance of the C─Co─N in HER application. Further, we have measured inductively coupled plasma‐optical emission spectrometry (ICP‐OES) to estimate the accurate cobalt and phosphorus contents. The estimated Co and P contents in the electrode before and after cyclic tests are given in Table S4 (Supporting Information). The Co content was decrased from 4.6% to 2.6%, whereas the P content decreased from 1.6% to 0.65%. It clearly showed that the good stability and durability of the fabricated C─Co─N electrode during HER application. The excellent stability and durability of the HCC electrocatalyst are mainly due to the protection of the catalytic materials (Co_2_P_2_O_7_ and Co_2_P) by a thick carbon shell, which restricted the leaching of catalytic materials from the HCC and prevented aggregation of the material during long‐term catalytic reaction. For comparison, we characterized the post‐HER stability of the Co─N electrocatalyst by FESEM and EDS mapping (Figure S21, Supporting Information). The porous 1‐D nanorod morphology of the Co─N product was lost and converted into aggregated particles‐like structures after the stability measurements. This indicates that the morphologies of the Co─N product were severely affected during the HER catalytic reaction for longer‐term operation due to the absence of a protecting carbon layer. However, the EDS mapping studies implied the existence of all expected elements in the particle‐like morphologies. The post‐HER analysis revealed that the as‐fabricated HCC was extremely stable for HER measurements and was useful for hydrogen generation. The excellent electrocatalytic properties for HER activity of C─Co─N modified electrodes are due to the following factors. i) The unique nitrogen doped carbon hollow structures provide good permeability, large active surface area, easy access to catalytic active sites, and enhanced electrical conductivity. ii) The presence of mixed cobalt products (Co_2_P_2_O_7_ & Co_2_P) enhanced catalytic performance compared to pure Co_2_P_2_O_7,_ and the cobalt phosphate‐based materials showed higher catalytic activity than the metallic cobalt due to enhanced adsorption and dissociation of water molecules over the P‐doped Co materials. iii) The well‐defined carbon shell over the Co_2_P_2_O_7_ and Co_2_P materials prevented leaching and aggregation of catalytic material the electrolysis, which provided superior stability for long‐term HER operation. iv) The carbon shell with high graphitization provided unique pore size and good electron transport properties, which enhanced the mass transport and reaction kinetics of the HER reaction. Therefore, the new HCC material fabricated through a simple and green approach can be a promising electrocatalytic material for HER applications.

### 2.3 Density Functional Theory (DFT) Calculation of Hollow Carbon Capsules (HCC)

**Figure 4:**
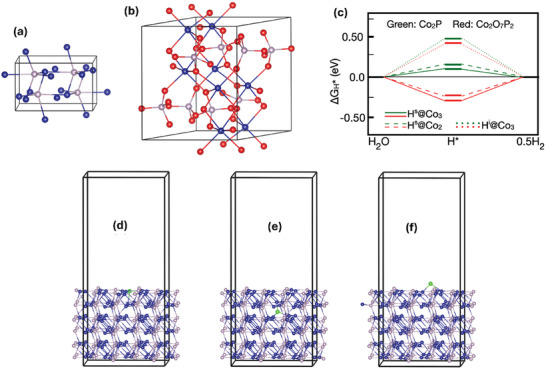
Ball and stick model of a) Co_2_P and b) Co_2_O_7_P_2_ structures. Blue, red, and gray color balls are Co, O, and P atoms, respectively. c) Calculated free energy of HER for different active sites in Co_2_P and Co_2_O_7_P_2_ and Ball and stick modes of Co_2_P_2_ surface with stable H adsorption at different places. d) H at surface of Co with three coordination, e) H at interstitial (inside the crystal) bonded with Co, and f) H at surface of Co with two coordination.

The obtained superior performance of the cobalt containing HCC electrocatalyst was further verified by DFT calculation (**Figure** **4**). The detailed experiments used for the DFT studies were provided in the supporting information. In the synthesized compounds, Co_2_P and Co_2_O_7_P_2_ were in orthorhombic (Space group: *pnma*, No: 62) and monoclinic (Space Group: *p*121, No: 14) crystal structures, respectively. These experimentally obtained structures and parameters were used for the DFT calculations to understand the HER. The surface structure was cleaved from the optimized crystal structures of Co_2_P and Co_2_O_7_P_2_ to study hydrogen adsorption and HER properties. We considered five active sites(H^s^@Co_2_, H^s^@Co_3_, H^i^@Co_3_, H^s^@P_3_, and H^i^@P_2_ such as two coordinated H at the surface of Co (H^s^@Co_2_), three coordinated H at the surface of Co (H^s^@Co_3_), three coordinated H at the interstitial Co (H^i^@Co_3_), three coordinated H at surface of P (H^s^@P_2_), two coordinated H at interstitial P (H^i^@P_2_) in both Co_2_P and Co_2_O_7_P_2_. Among five these identified sites, P‐based sites were unable to form bonds with an H atom compared to Co‐sites. The calculated free energies of the HER at the interstitial sites, which are located inside the crystals, exceeded 0.4 eV. In contrast, surface active sites with two and three coordinated Co exhibited free energies within the range of ±0.25 eV. This observation indicates that interstitial sites are unstable for the HER compared to surface sites. Moreover, while Co_2_P exhibited desorption conducive to HER, Co_2_O_7_P_2_ demonstrated an adsorptive nature for the HER.

### 2.4 Fabrication of an AEM Water Electrolyzer

**Figure 5:**
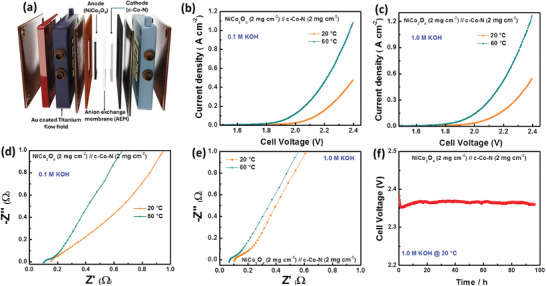
a) Schematic illustration of AEM water electrolyzer assemble setup with loading mass of 2 mg of NiCo_2_O_4_ (as anode) and 2 mg of C─Co─N (as cathode), LDPE‐VBC‐TMA used as AEM membrane. Linear sweep voltammogram of fabricated AEM water electrolyzer recorded at different concentration of KOH electrolyte b) 0.1 m KOH, c) 1.0 m KOH at 20 and 60 °C. Electrochemical impedance spectra of the device d) 0.1 m KOH, e) 1.0 m KOH at 20 and 60 °C. f) Stability of the AEM device recorded by chronopotentiometry at applied current density of 500 mA cm^−2^ for 96 h in 1.0 m KOH at 20 °C.

Based on the improved HER performance of the fabricated C─Co─N electrocatalyst, we further investigated an AEM single‐cell water electrolyzer. The C─Co─N electrocatalyst was used as a cathode, and a commercial spinal oxide of NiCo_2_O_4_ was used as the anode catalyst. Radiation‐grafted low‐density polyethylene (LDPE)‐vinylbenzyl chloride (VBC)‐trimethyl amine (TMA) membrane was used as anion exchange membrane.^[^ ^50^ ^]^ The fabrication and testing of AEM process was explained in the supporting information. The single‐cell AEM water electrolyzer performance was investigated at temperatures of 20 and 60 °C with feed electrolyte of 0.1 and 1.0 m KOH. **Figure** **5**a shows the schematic diagram of the fabrication of the AEM water electrolyzer (WE). Figure 5b,c shows the polarization curves of the AEM water electrolyzer with 1.0 and 0.1 m KOH feed electrolyte at 20 and 60 °C. As shown, the AEM water electrolyzer achieved a current density of 10 mA cm^−2^ at 1.88 and 1.71 V at 20 and 60 °C respectively. A low area specific resistance (ASR) in the AEM water electrolyzer was more favorable for electrolyzer performance, calculated from Figure 5d,e. Further increasing the temperature (20 – 60 °C) reduced the ASR from 0.274 to 0.186 Ω cm^2^. Further, we investigated AEM water electrolyzer performance with 1.0 m KOH temperatures of 20 and 60 °C. These results show that the AEM water electrolyzer achieved a current density of 10 mA cm^−2^ at 1.81 and 1.61 V at 20 and 60 °C, respectively. In contrast, the AEM water electrolyzer achieved a current density of 500 and 1000 mA cm^−2^ at cell voltages of 2.19 and 2.33 V with 1.0 m KOH at 60 °C and Table S3 (Supporting Information) shows a comparison of AEM water electrolyzer performance with that of C─Co─N electrocatalyst and literature reports. This confirmed that the ASR decreased to 0.126 from 0.189 Ω cm^2^ (at 20 and 60 °C) due to improved reaction kinetics at high temperature, and the results are presented in Figure 5d,e). To investigate the stability of the cathode electrocatalyst in an AEM water electrolyzer, we performed a stability test of the AEM using chronopotentiometry at 500 mA cm^−2^ with 1.0 m KOH at 20 °C for 96 h, and the results are presented in Figure 5f. The initial decrease in potential was ≈2.353 V at 2.5 h, which slightly increased to 2.367 V and then decreased to 2.360 V at 96 h. This confirm the good stability of C─Co─N electrocatalyst in a cathode due to the encapsulation of CoP nanoparticles in a nitrogen‐ containing graphitic carbon layer and hollow carbon capsules to improve the surface area of an electrocatalyst to achieve improved performance.^[^ ^51^, ^52^ ^]^

## 3 Conclusion

We demonstrated a simple method for the fabrication of 1D Co‐MOF nanorods through a green approach using a mixture of water and ethanol as solvent without any templates, hazardous materials, or tedious processes. The obtained Co‐MOF was successfully utilized as a self‐degraded template for the fabrication of highly uniform HCC containing Co‐based electrocatalyst (Co_2_P_2_O_7_ & Co_2_P). The HCC electrocatalyst demonstrated remarkable HER performance including a low overpotential (137 mV @ η_10_), high Tafel slope (85.5 mV dec^−1^), and excellent long‐term stability compared to other Co‐based electrocatalyst. This work provides a new direction for designing various transition metal‐based hollow nanomaterials with different morphologies using metal phosphonates as self‐degraded templates for the fabrication of advanced mixed transition metals as multifunctional catalysts in energy storage and harvesting applications.

## 4 Experimental Section

### Materials

Cobalt (II) acetate (CA; Co(CH_3_COO)_2_∙4H_2_O), Nafion (5% in alcohols and water), dopamine, and phenylphosphonic acid (PPA; C_6_H_5_P(O)(OH)_2_, 98%,) were obtained from Sigma–Aldrich. Ethanol (absolute, 99%) and methanol (99.8%) were purchased from Fisher Chemical and Junsei Chemical, respectively. Tris HCL (pH 8.5) was obtained from Biosolution Co. Ltd., South Korea. Deionized (DI) water (resistivity: 18.2 MΩ, Elga DI water system) was used for the preparation of all aqueous solutions.

### Preparation of 1‐D Cobalt‐MOF Nanorods

The 1‐D Co‐MOF nanorods were prepared by a hydrothermal method, as depicted in Figure 1a. The initial precursors (440 mg CA and 280 mg; PPA) were mixed with 70 mL of a DI water/ethanol (1:1) mixture under vigorous stirring. The mixed solution was poured into a Teflon‐lined autoclave and treated at 120 °C for 8 h. Finally, the obtained pink cobalt phenylphosphonate (Co‐MOF) precipitate was collected by vacuum filtration, dried in a vacuum oven at 50 °C, and named Co‐MOF (Figure S1a, Supporting Information).

### Preparation of Polydopamine Coated 1‐D Co‐MOF Nanorods (DP‐Co‐MOF)

The as‐prepared Co‐MOF (200 mg) was dispersed in Tris‐HCl solution (80 mL) with using a probe sonicator for 30 min. Then freshly prepared dopamine in Tris‐HCl solution was added immediately into the Co‐MOF dispersion under stirring. The reaction continued for 12 h at room temperature (Figure 1a). The polydopamine‐coated Co‐MOF was collected by centrifugation and washed with water and ethanol several times. The obtained dark brown polydopamine coated Co‐MOF was denoted as DP‐Co‐MOF (Figure S1b, Supporting Information).

### Preparation of Hollow Carbon Capsules from DP‐Co‐MOF

The as‐synthesized DP‐Co‐MOF was treated at 800 °C for 2 h under a N_2_ atmosphere to form hollow carbon capsules, and the resulting product was denoted as C─Co─N (Figures 1a and S1d, Supporting Information). Further, the same DP‐Co‐MOF was treated in air (800 °C for 3 h) and referred to as as C─Co─A (Figure S1f, Supporting Information). For comparative studies, Co‐MOF was also treated under both air (800 °C for 3 h) and N_2_ (800 °C for 2 h) atmospheres, and the resulting products were referred to as Co─A (Figure S1e, Supporting Information) and Co─N (Figure S1c, Supporting Information), respectively.

## Conflict of Interest

The authors declare no conflict of interests.

## Supporting information



Supporting Information

## Data Availability

The data that support the findings of this study are available from the corresponding author upon reasonable request.
