# Supplementary material for: A Simple and Efficient Non‐Noble Cathode Catalyst Based on Carbon Hollow Nanocapsules Containing Cobalt‐Based Materials for Anion Exchange Membrane Water Electrolyzer
Source: Small. 2025 Feb 3;21(9):2411019. doi: 10.1002/smll.202411019 (PMC11878260; doi:10.1002/smll.202411019)
Supplement: Supplementary file 1 — Supporting Information [file SMLL-21-2411019-s001.docx]

**Supporting Information**

A simple and efficient non-noble cathode catalyst based on carbon hollow nanocapsules containing cobalt-based materials for anion exchange membrane water electrolyzer

Sivaprakasam Radhakrishnan^1^, Shanmugam Ramakrishnan^2^, Santhosh Kumar Jayaraj^3^, Mohamed Mamlouk^2^*, Byoung-Suhk Kim^3,4^*

Dr. Sivaprakasam Radhakrishnan^1^,

^1^Department of Chemistry, School of Sciences and Humanities, SR University, Warangal 506371, Telangana, India

Dr. Shanmugam Ramakrishnan^2^, Prof. Mohamed Mamlouk^2^

^2^School of Engineering, Newcastle University, United Kingdom

E-mail: mohamed.mamlouk@ncl.ac.uk

Dr. Santhosh Kumar Jayaraj^3^, Prof. Byoung-Suhk Kim^3,4^
^3^Department of Organic Materials & Textile Engineering, Jeonbuk National University, 567 Baekje-daero, Deokjin-gu, Jeonju-si, Jeollabuk-do 54896, Republic of Korea.

^4^Department of JBNU-KIST Industry-Academia Convergence Research, Jeonbuk National University, 567 Baekje-daero, Deokjin-gu, Jeonju-si, Jeollabuk-do 54896, Republic of Korea.

E-mail: kbsuhk@jbnu.ac.kr

**Materials characterization**

Field emission scanning electron microscopy (FESEM, Carl Zeiss-SUPRA40V) and high-resolution transmission electron microscopy (HR-TEM, Tecani GSF205-TWIN) were used to study the morphological features of the as-derived Co-based nanomaterials. The as-obtained Co-based product crystal characteristics was confirmed by XRD (Rigaku, Japan). The surface area of the HCC product was verified by Brunauer-Emmett-Teller (BET; Belsorp analyzer) process under nitrogen adsorption/desorption method. Raman spectroscopy was recorded for prepared materials using a HORIBA Raman spectroscopy. The chemical composition and their oxidation states were confirmed by X-ray photoelectron spectroscopy (XPS; VG Multilab 2000) The extended X-ray absorption fine structure (EXAFS) and X-ray absorption near edge structure (XANES) measurement at the Pt-k edges were obtained using Sc-detection (model R-XAS, Riagaku, Japan) with total electron yield detection. An inductively coupled plams optical emission spectrometer (ICP-OES) using Thermo Fisher Scientific (Model No. iCAP 7400 Duo).

**Electrochemical characterization of hydrogen evolution reaction (HER) performance**

The electrochemical studies such as cyclic voltammetry (CV), linear sweep voltammetry (LSV), chronoamperometry (CA), and electrochemical impedance spectroscopy (EIS) were measured using Versastat 4 electrochemical workstation under three-electrode configuration using Co-based nanomaterials modified nickel foam electrode (1 cm × 1 cm), a graphite rode, and Ag/AgCl (saturated KCl) as working, counter and refence electrodes, respectively. The catalytic ink was prepared for the modification of nickel foam as follows, as-obtained Co-based nanomaterials (5 mg) was added into the solution (mixture of isopropanol (0.7 mL), DI water (0.25 mL) and Nafion (50 µL)) under sonication up to fine dispersion. All the electrochemical measurements were performed using 1.0 M KOH solution (pH 13.6). The reported potential was changed to reversible hydrogen electrode (RHE) potential using the formula (*E*_RHE_ = *E*_Ag/AgCl_ + 0.059 × pH +0.197 V). LSV recorded at applied sweeping scan rate of 5 mV s^-1^. Potentials were *iR*-adjusted (where *i* and *R,* are current, electrolyte resistance, respectively). The electrochemical impedance spectroscopy was collected in the frequency region from 100 kHz to 0.1 Hz at the dc potential corresponding to η10. The stability of the measurement was performed by CA method at a potential corresponding to a current density of 100 mA cm^-2^.

**Computational methods**

DFT calculations were performed using the Vienna Ab-initio Simulation package (VASP)to understand hydrogen evolution reaction in Co_2_P and Co_2_O_7_P_2_^S1^. The projector augmented wave (PAW) was used to describe the valance electrons of Co, P, O, and H atoms^S2,S3^. The revised Perdew– Burke–Ernzerhof (RPBE) functional was used to correct the exchange-correlation functional part^S4^.

A 4×6×6 and 3×3×2 *k*-grid is used to optimize the structure and for calculating electronic structure, respectively. 500 eV of cut-off energy is supplied. Self-consistent field calculations are used to optimize electronic and ionic steps until the energy difference between two successive steps becomes 0.01 meV and the force constant on each ion reaches to 0.01 eV/Å, respectively. The H adsorption free energy is calculated from $\Delta G_{H*}=E_{sur+H}-E_{sur}-0.5\times E_{H_{2}}+\Delta ZPE-T\Delta S$, where $E_{sur+H}$, $E_{sur}$, $E_{H_{2}}$, *T*, and Δ*S* are energy of hydrogenated surface slab, pure surface slab, and hydrogen molecule, change in zero point energy, absolute temperature, and entropy change of hydrogen adsorption at 300K, respectively. For, Ni based material, this is calculated using $\Delta G_{H*}=E_{sur+H}-E_{sur}-0.5\times E_{H_{2}}+0.24$ eV^S5^.

**Fabrication of AEM water electrolyzer (AEMWE)**

The anode catalyst NiCo2O_4_ were mixed in tetrahydrofuran (THF), and Styerene-ethylene-butylene-styrene (SEBS) ionomer and polytetrafluoroethylene (PTFE) was employed as binder to make the coating solution. The prepared coating solution was sprayed (2 mg cm^-2^) using a spray gun on a titanium fiber felt gas-diffusion electrode (GDL). Under identical conditions were followed for the fabrication of cathode material (c-Co-N). The cathode ink was sprayed on carbon GDL with an optimized platinum loading of 0.2 mg_pt_ cm^-2^. After fabricating the respective GDLs, the anode and cathode catalysts were immersed into the trimethyl amine (TMA) for 24 hrs. Subsequently, the fabricated electrode was washed with water several times to remove the impurities and dried at room temperature. Importantly, the anode, cathode and membrane components were treated with 1.0 M KOH before assembling the MEA. At last, the assembled MEA was kept in a water electrolyzer cell assembly and was torqued to 1.2 Nm. The electrolyzer cell was fed with 1.0 M and 0.1 M KOH electrolyte solution with a 50 mL/min flow rate, and measurement were conducted.

**Electrochemical analysis of AEMWE**

The linear sweep voltammetry (LSV) was obtained from 1.2 to 2.5 V at scan rate of 1 mV s^-1^, and electrochemical impedance spectroscopy (EIS) was performed from 100 kHz to 100 mHz with an amplitude of (V_rms_) 10 mV and cell voltage of 1.5 V. The AEM study was performed on a Gamry interface 5000E potentiostat.

**
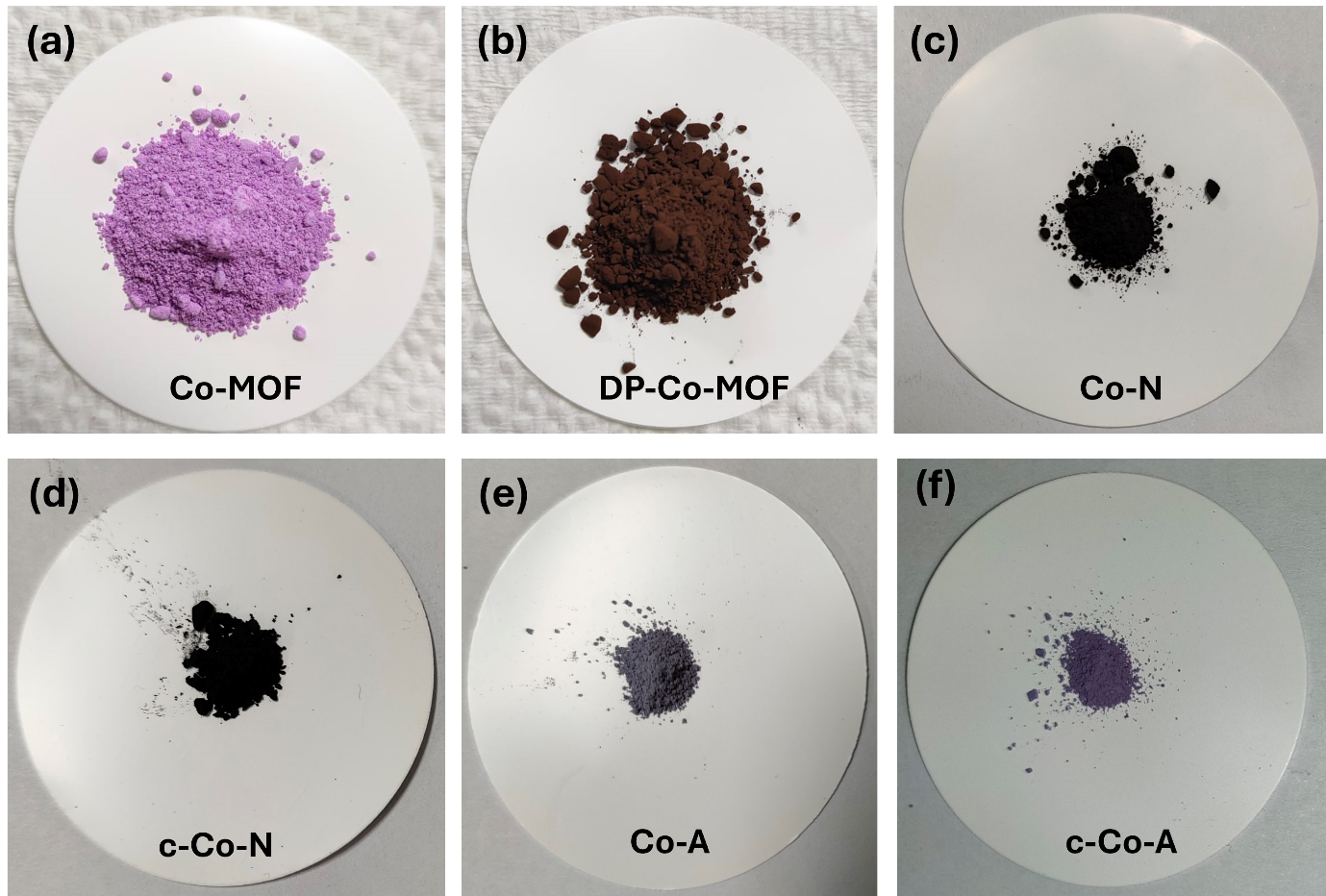
**

**Fig. S1.** Photographic images of as-synthesized various Co-based materials.

**
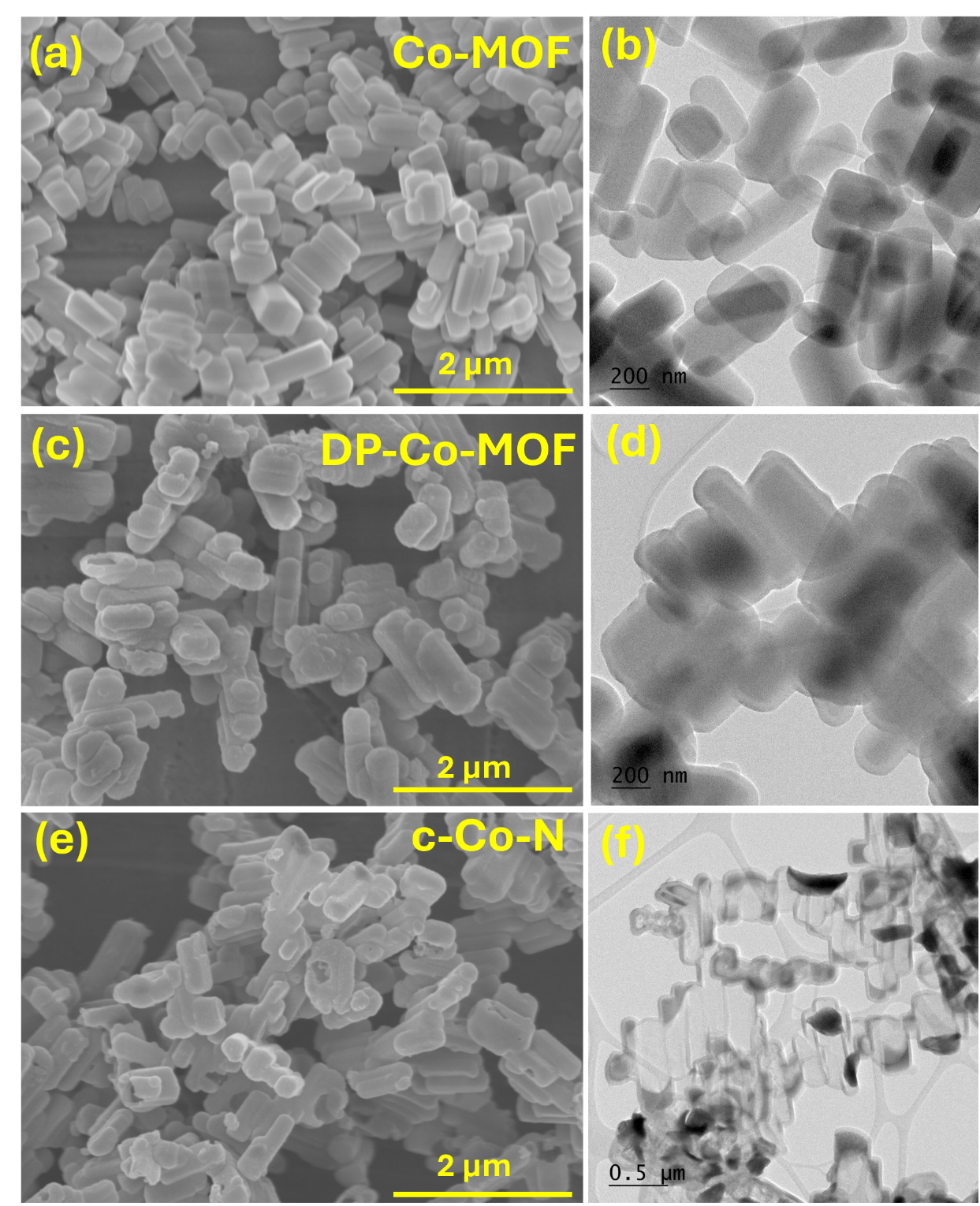
**

**Fig. S2.** FE-SEM and TEM images of Co-MOF (a, and b), DP-Co-MOF (c, and d) and c-Co-N (e and f)

**
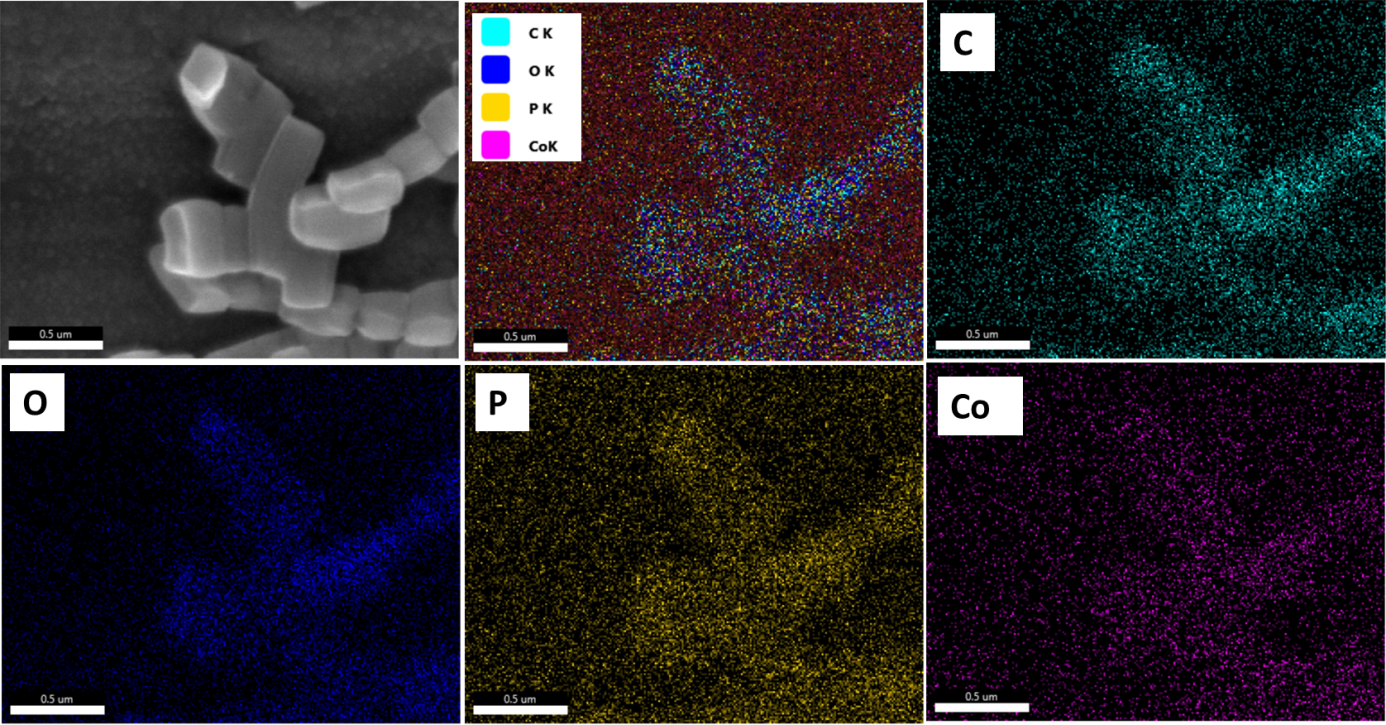
**

**Fig. S3.** Elemental mapping of Co-MOF.


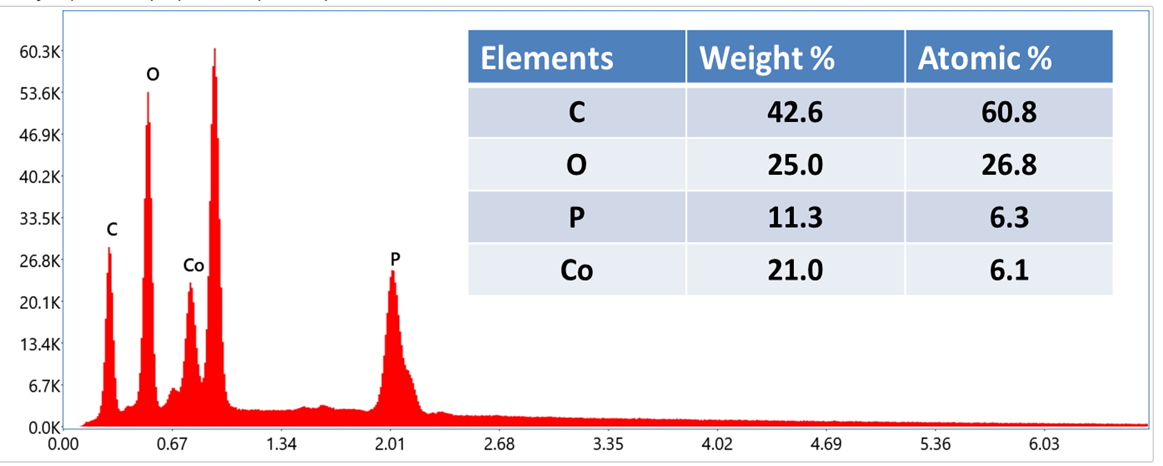


**Fig. S4.** EDX spectrum of Co-MOF





**Fig. S5.** XRD spectrum of Co-MOF


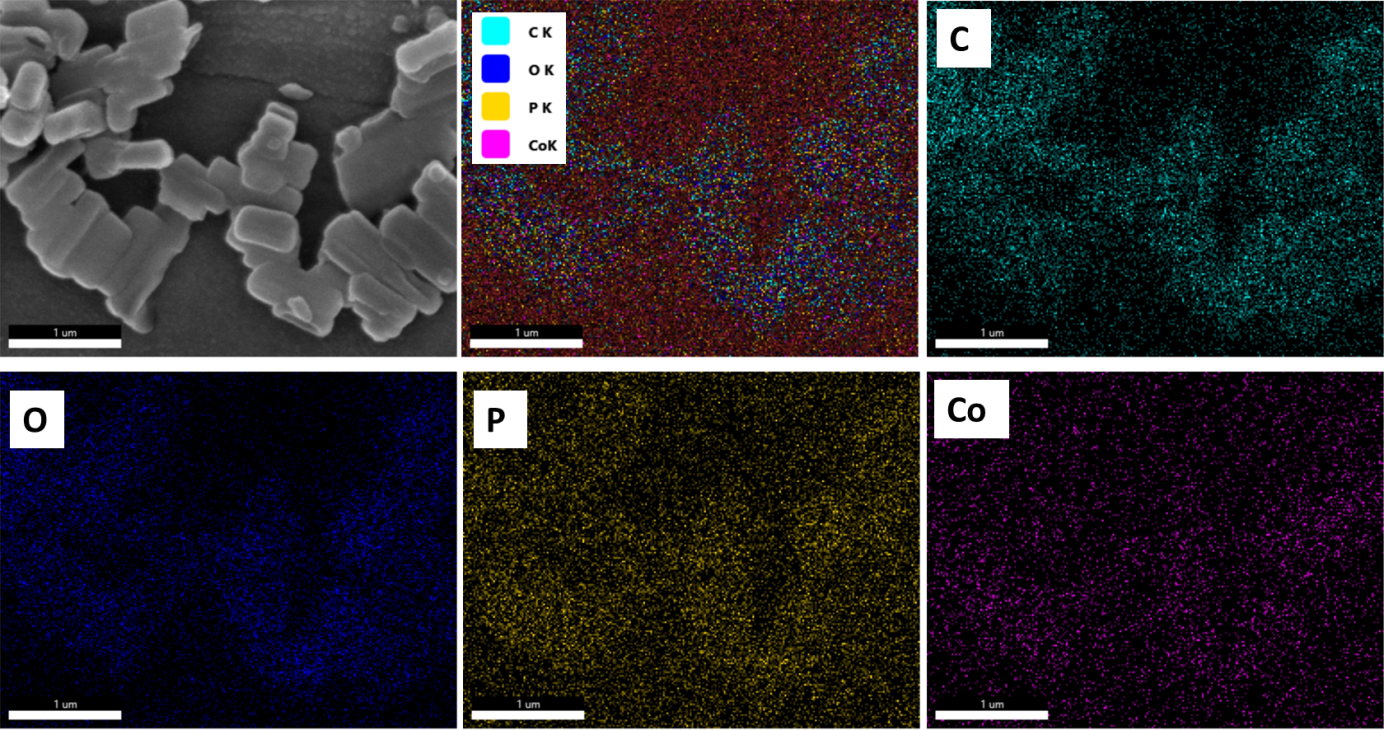


**Fig. S6.** Elemental mapping of DP-Co-MOF





**Fig. S7.** XRD spectrum of DP-Co-MOF


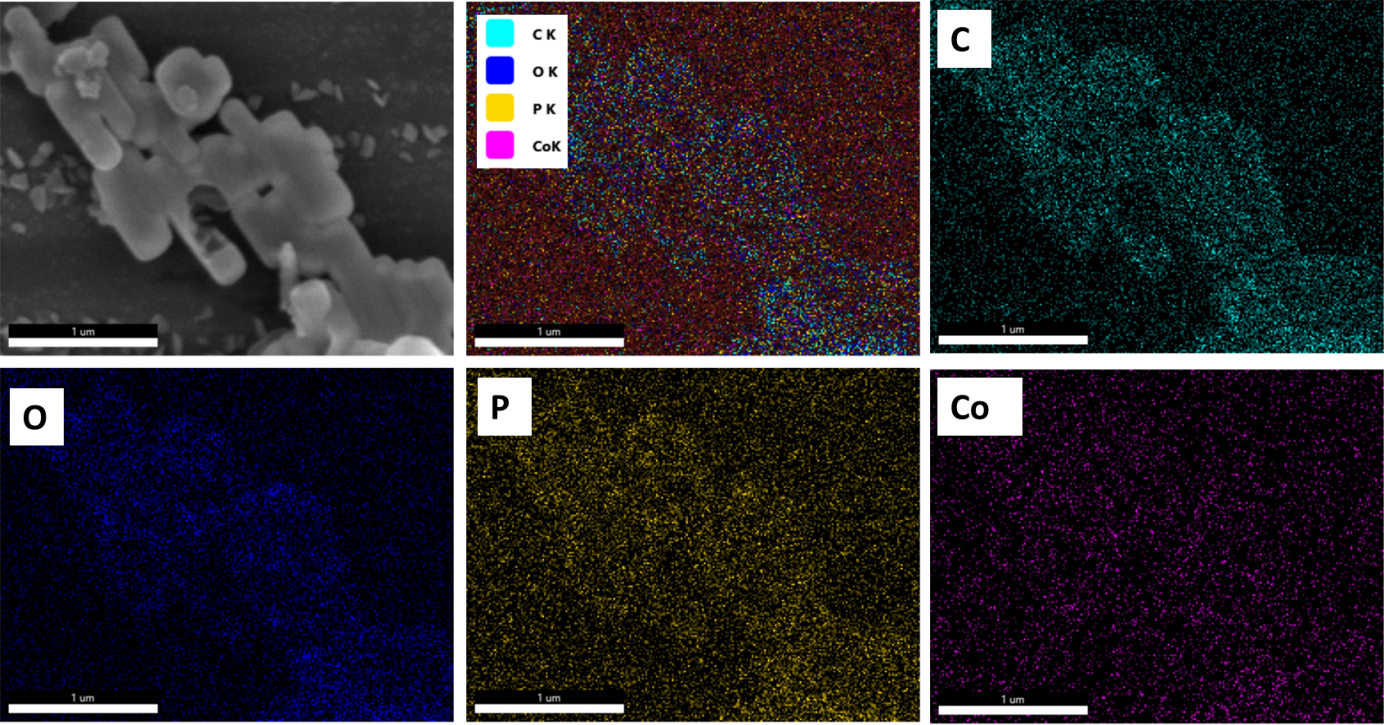


**Fig. S8.** Elemental mapping of c-Co-N

**
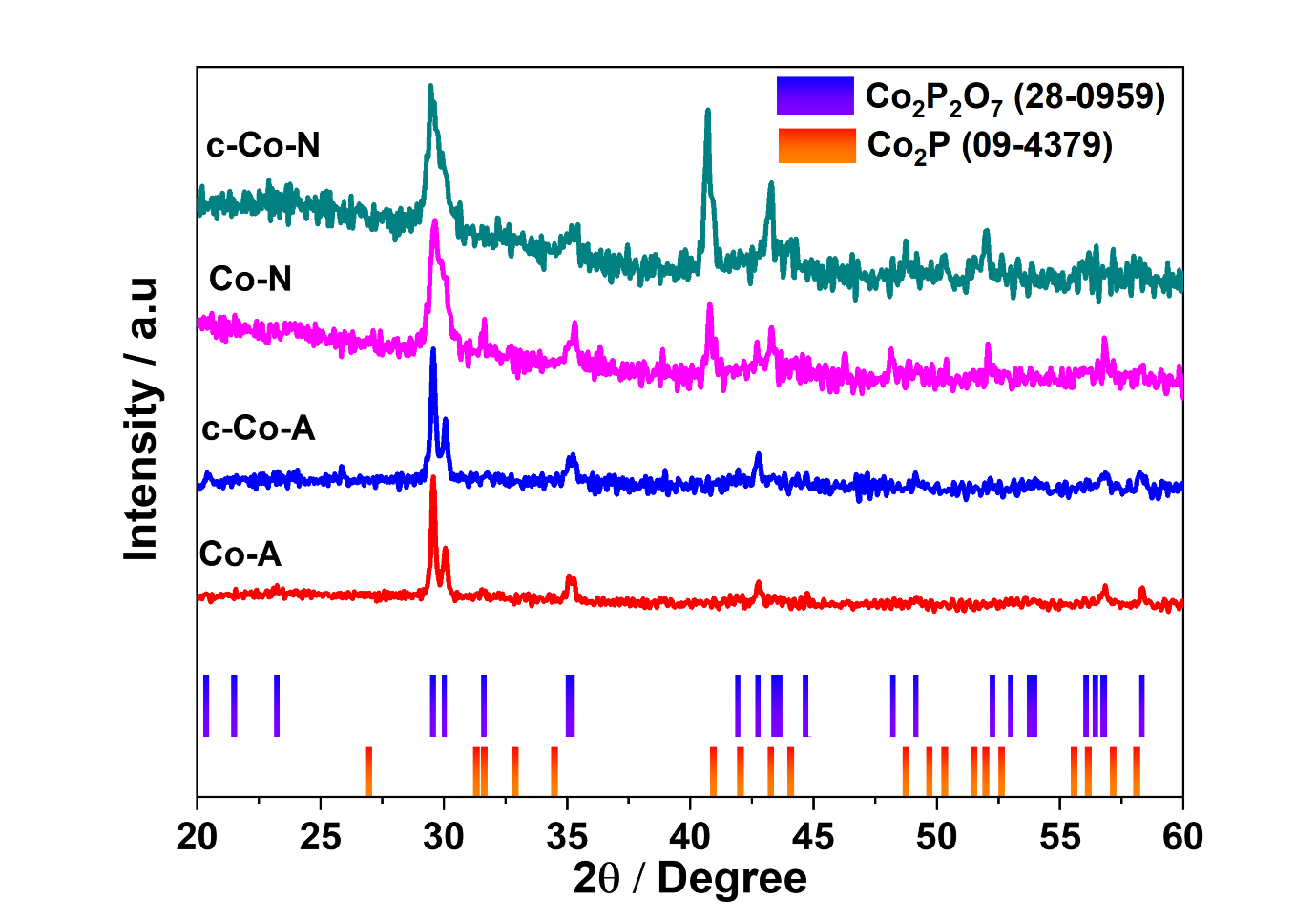
**

**Fig. S9.** XRD spectra of Co-A; c-Co-A, Co-N; and c-Co-N


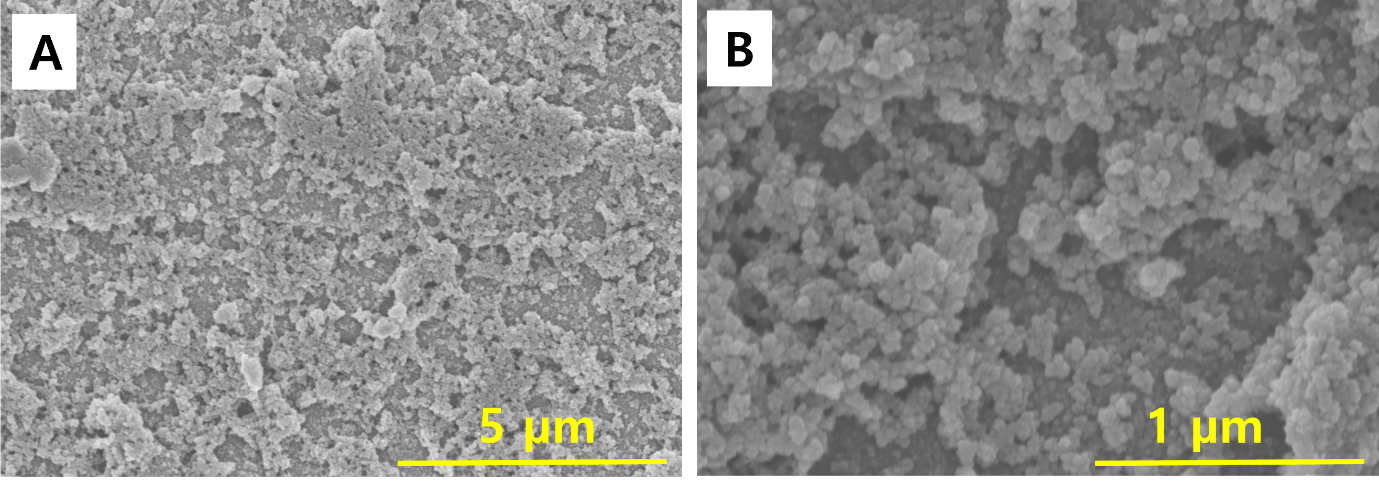


**Fig. S10.** FE-SEM images of c-Co-A


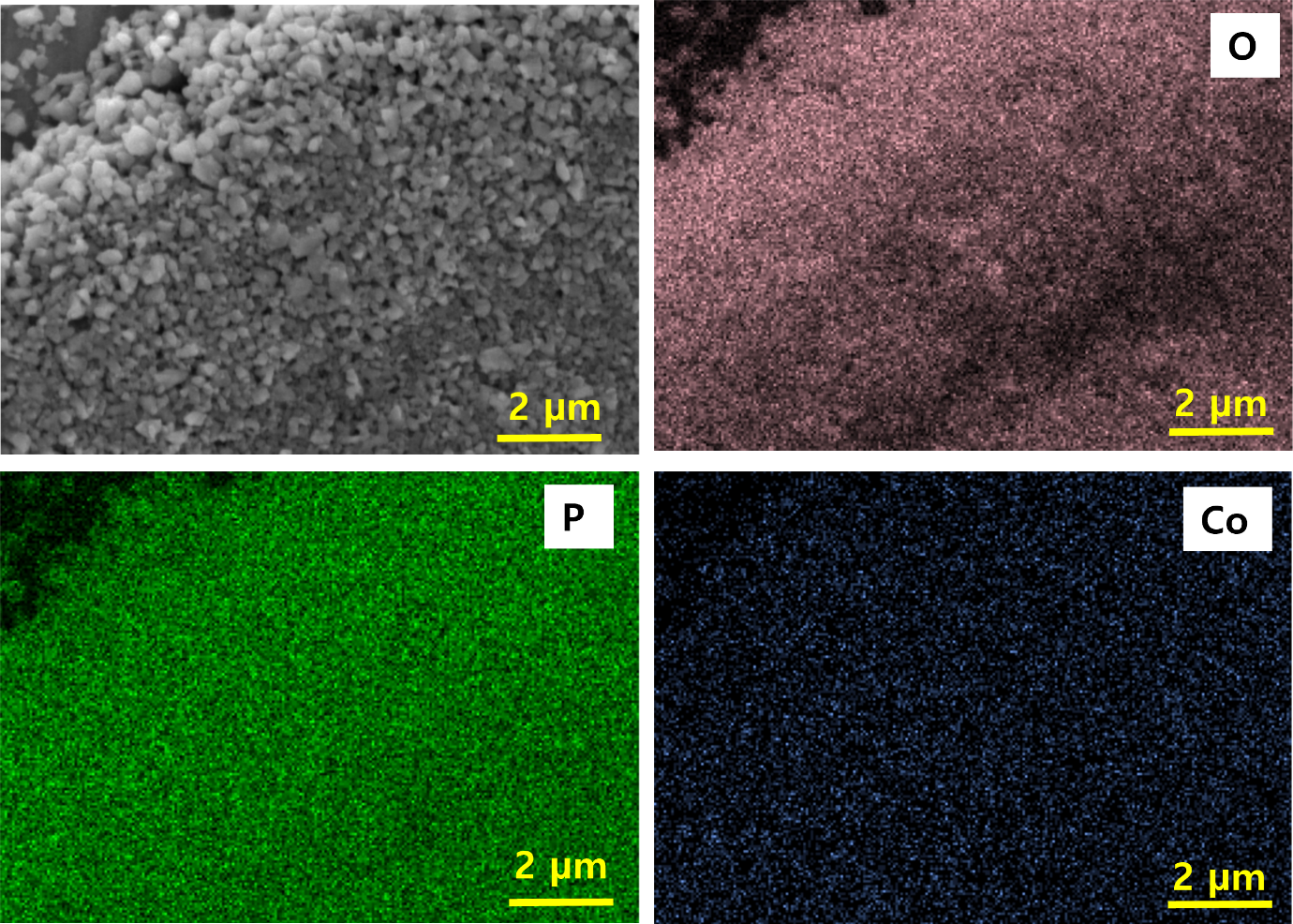


**Fig. S11.** Elemental mapping of c-Co-A


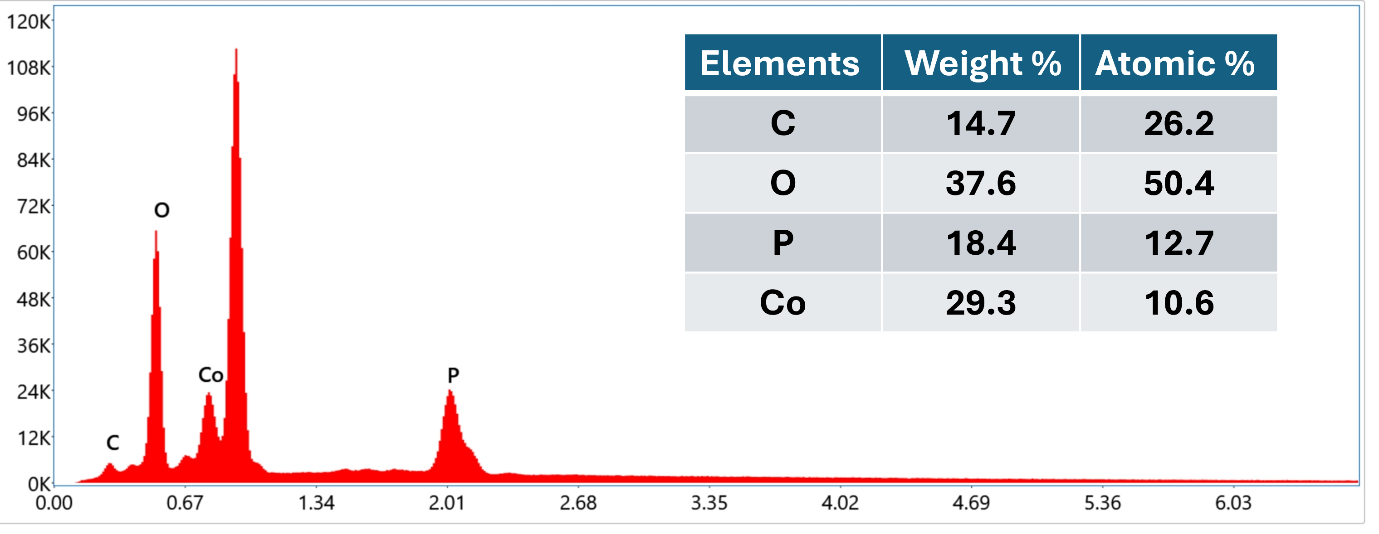


**Fig. S12.** EDX spectrum of c-Co-A


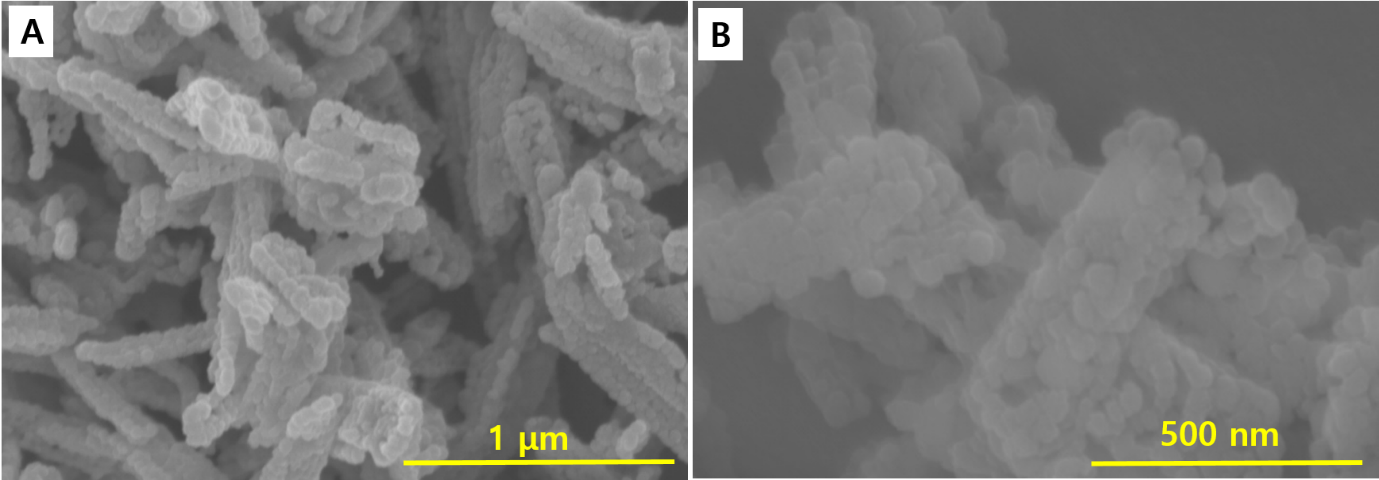


**Fig. S13.** FE-SEM images of Co-N


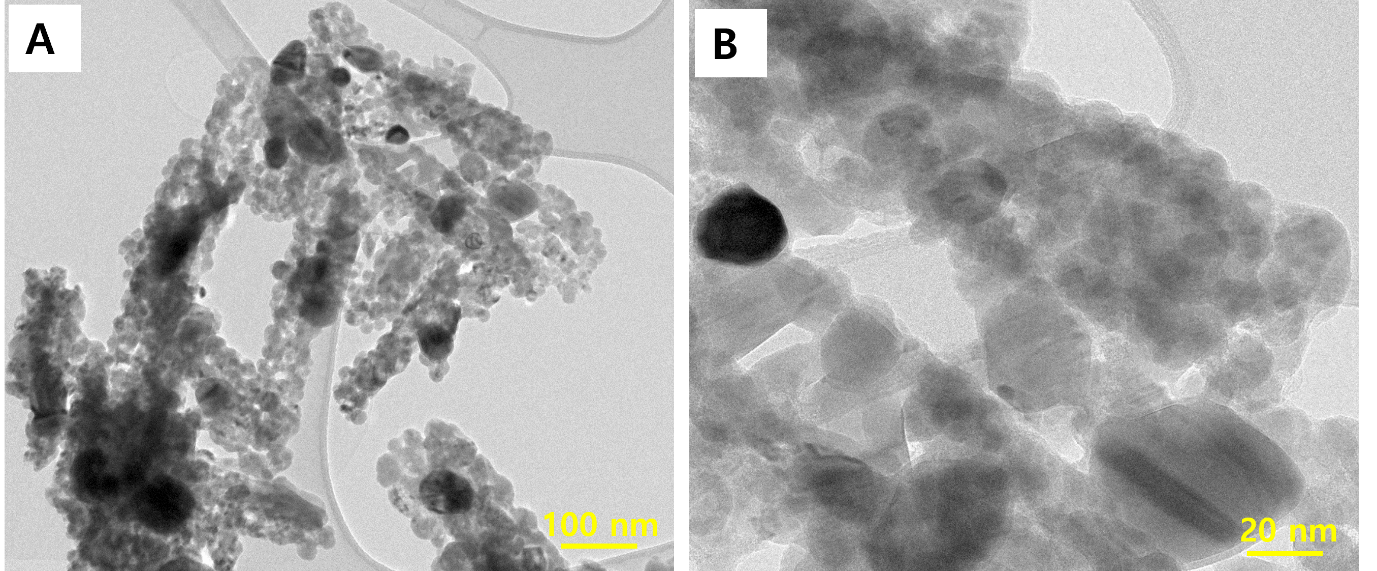


**Fig. S14.** TEM images of Co-N


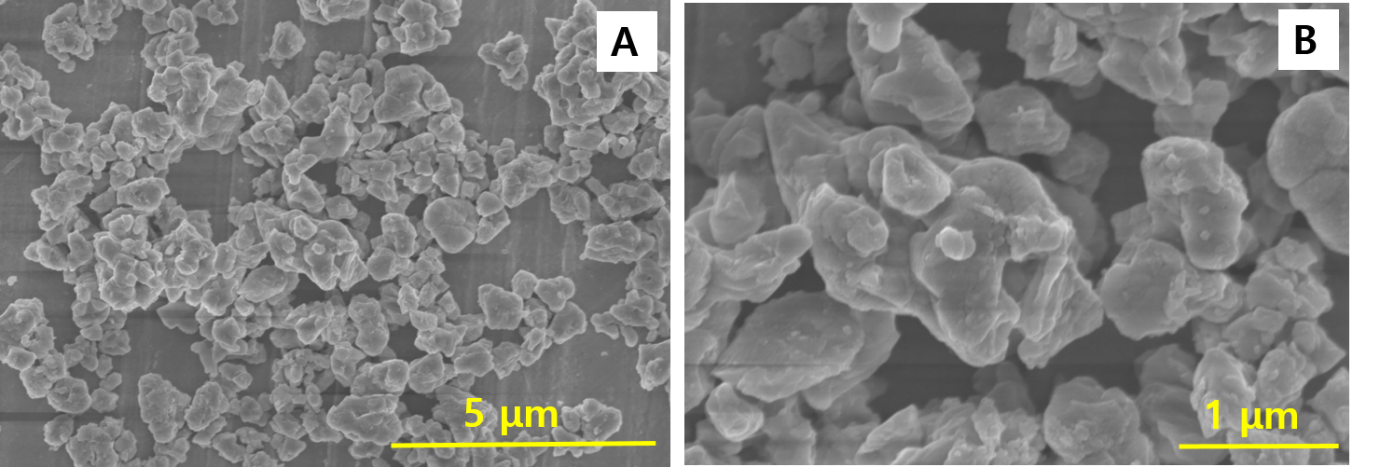


**Fig. S15.** FE-SEM images of Co-A


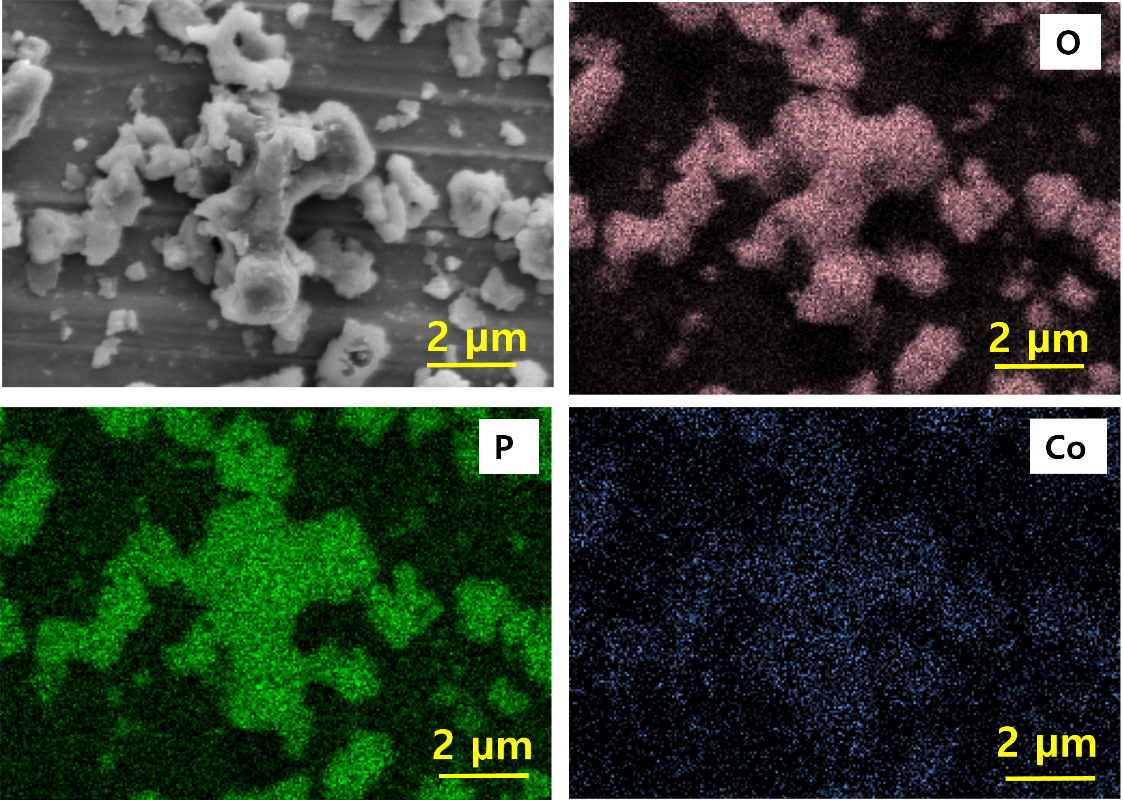


**Fig. S16.** Elemental mapping of Co-A


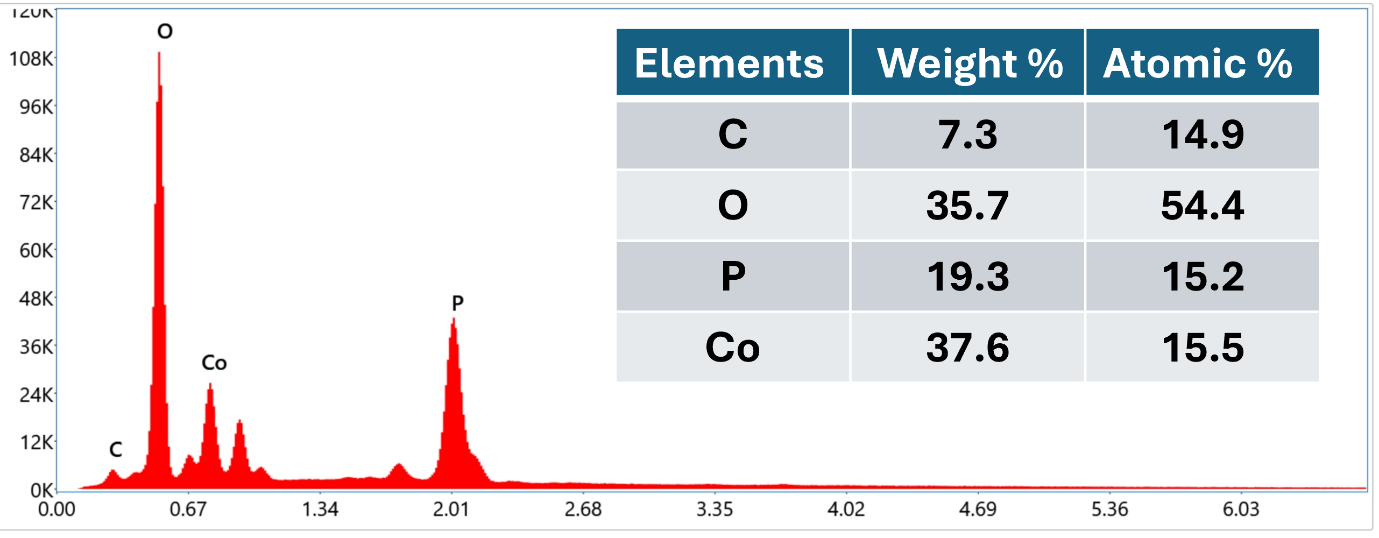


**Fig. S17.** EDX spectrum of Co-A

**
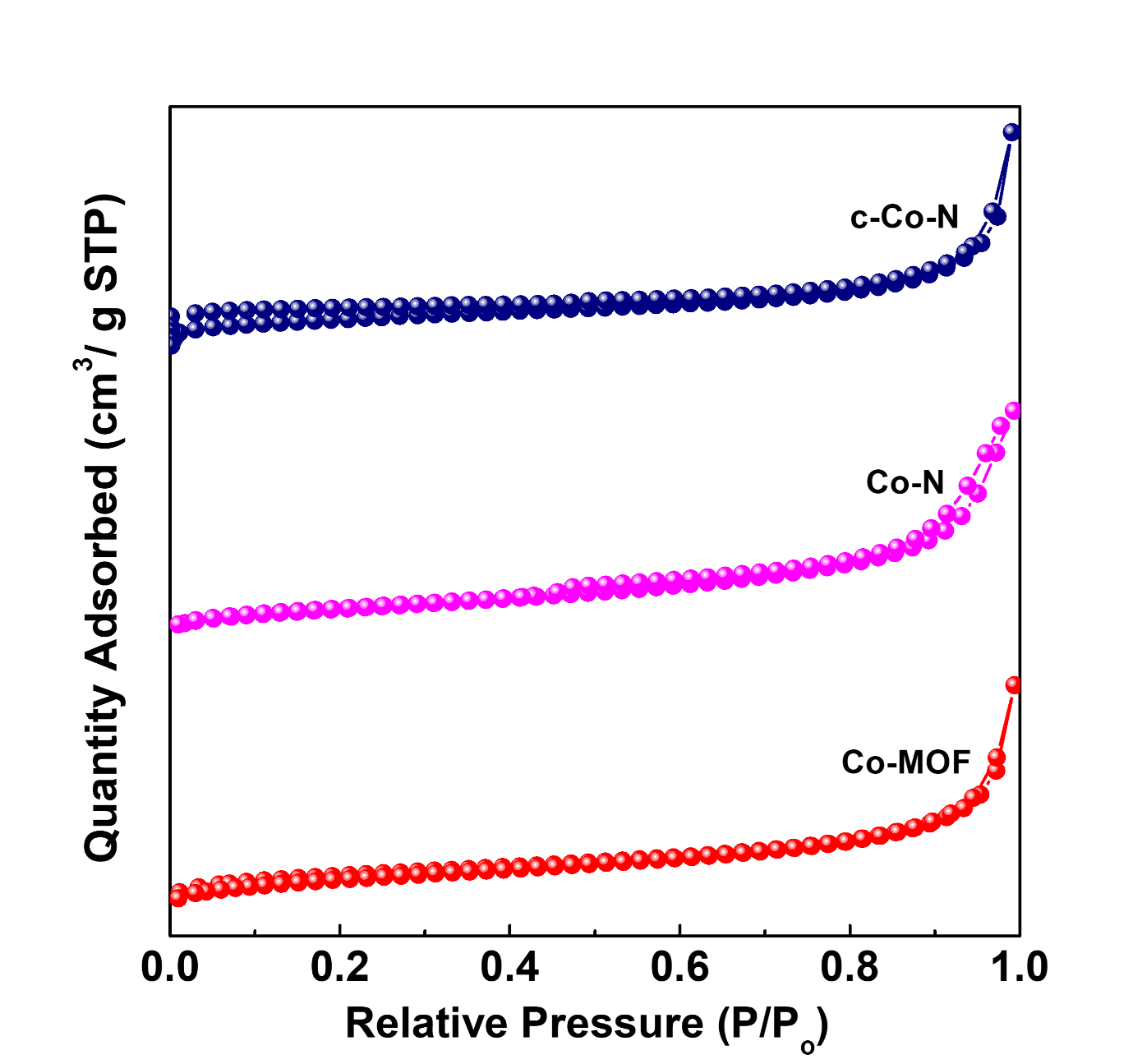
**

**Fig. S18.** N_2_ adsorption/desorption isotherms of Co-MOF, Co-N and c-Co-N.


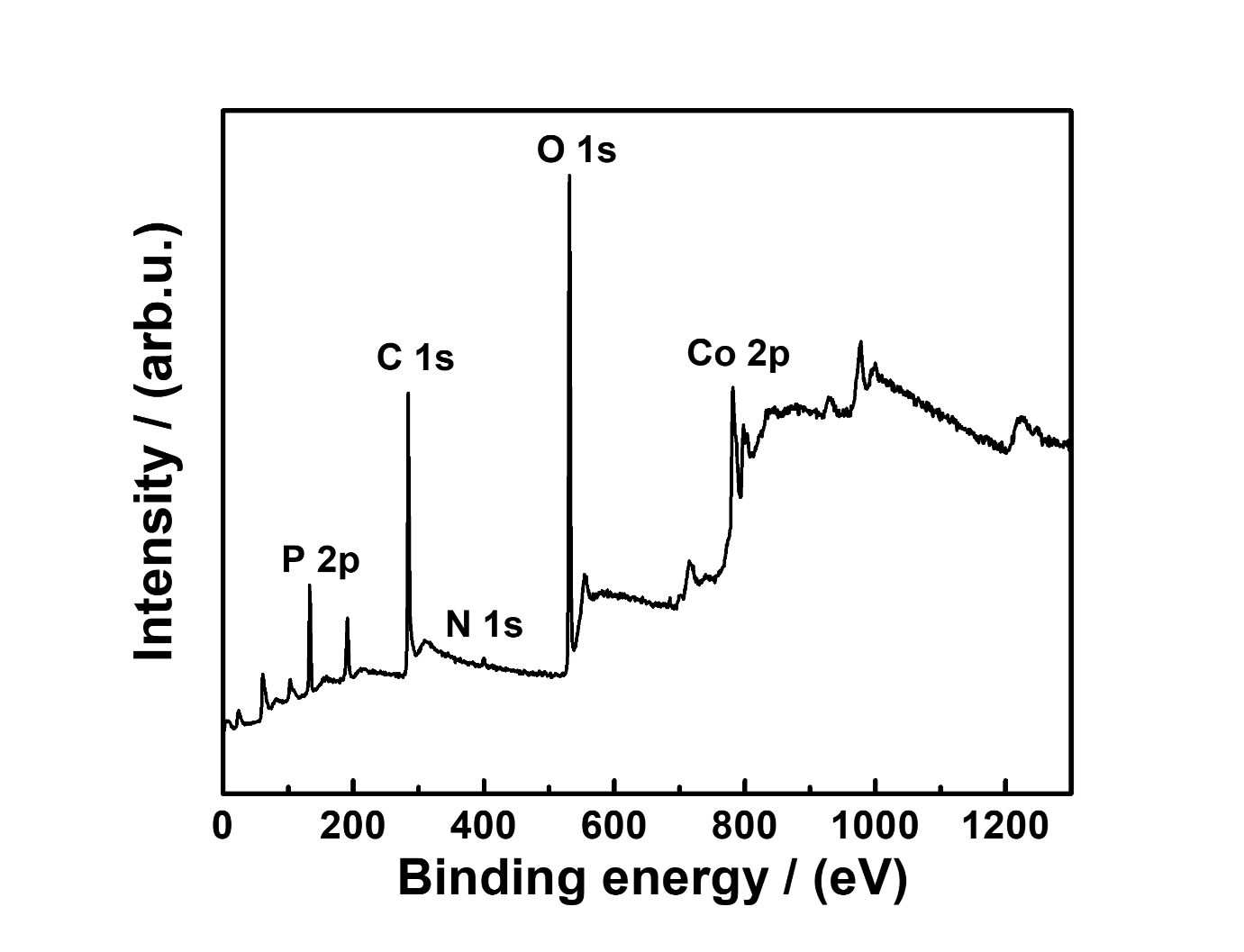


**Fig. S19.** XPS survey spectrum of c-Co-N product


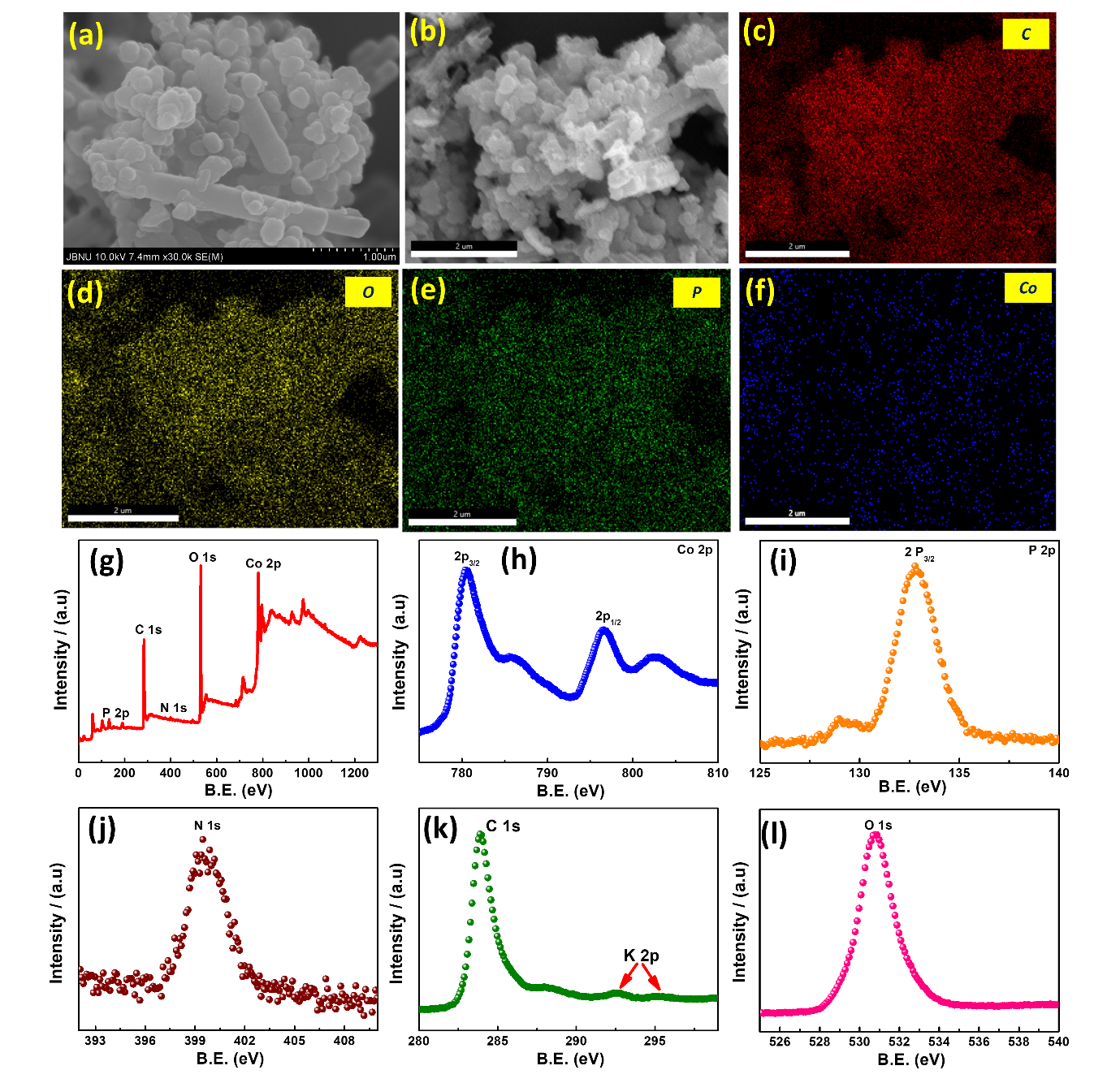


**Fig. S20.** (a) FE-SEM image (b-f) EDS mapping measurements and (g - l) XPS investigation of post HER c-Co-N product.


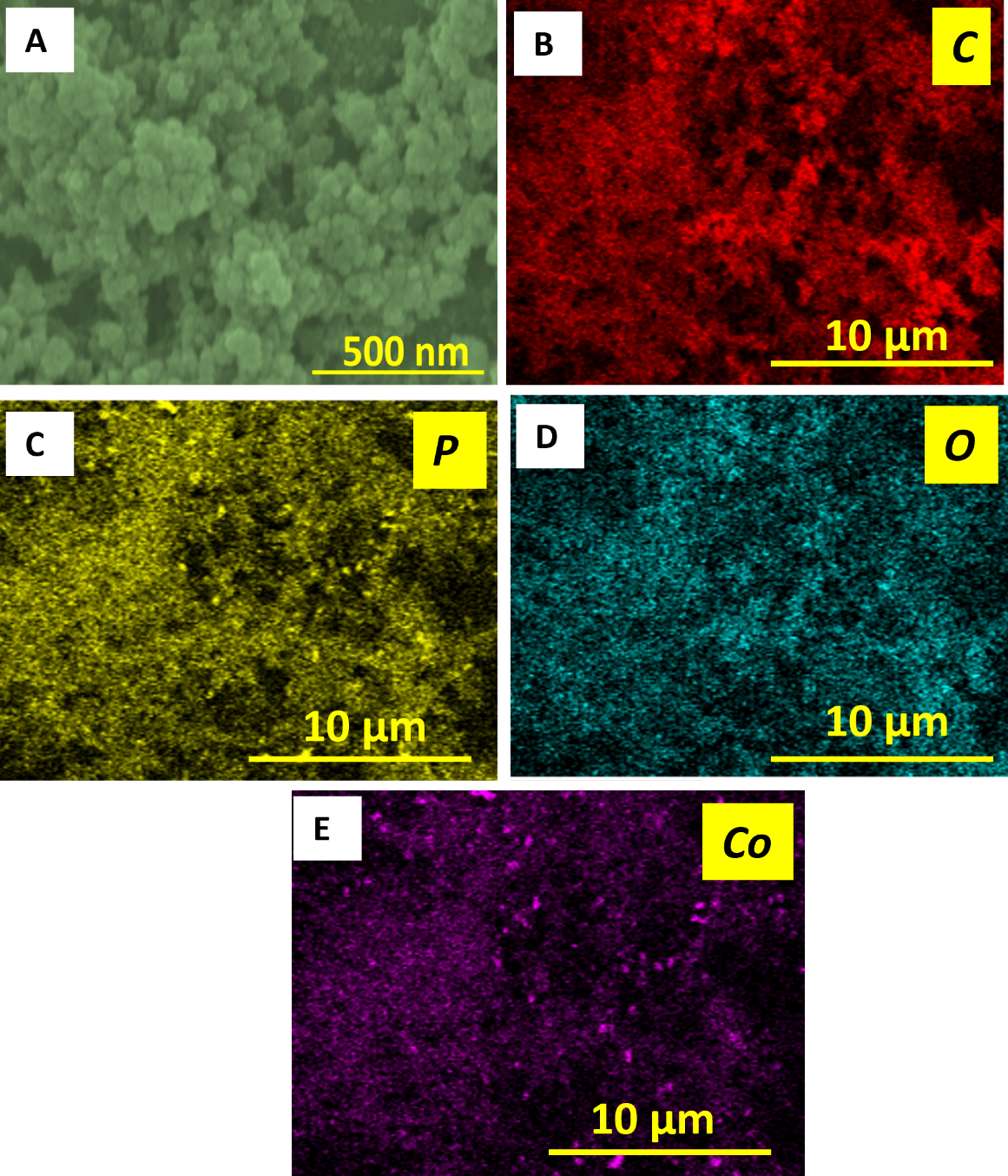


**Fig. S21.** FESEM and EDS mapping measurements of post HER Co-N material.

**Table S1.** XPS elemental composition of c-Co-N for post and pre-HER characterization

| **Name** | **Peak Binding Energy** | | **FWHM / eV** | | **Area (P) CPS** | | **Atomic / %** | |
| --- | --- | --- | --- | --- | --- | --- | --- | --- |
|  | **Pre-HER** | **Post-HER** | **Pre-HER** | **Post-HER** | **Pre-HER** | **Post-HER** | **Pre-HER** | **Post-HER** |
| C | 283.92 | 283.92 | 1.31 | 1.47 | 185157 | 164006.3 | 45.19 | 43.71 |
| Co | 781.54 | 780.57 | 4.69 | 3.81 | 360420 | 662753.2 | 7.21 | 14.46 |
| P | 133.32 | 132.82 | 1.85 | 2.36 | 67448.5 | 20934.16 | 11.11 | 3.76 |
| O | 531 | 530.79 | 1.93 | 1.98 | 348804 | 335907 | 35.22 | 37.03 |
| N | 400.45 | 399.62 | 2.52 | 2.58 | 8090.86 | 6073.27 | 1.27 | 1.04 |

**Table S2.** Comparison of HER catalytic properties of the HCC modified electrode with reported Co-based electrodes

| **Electrode material** | **Overpotential / mV at 10 mA cm^-2^** | **Tafel slope / mV dec^-1^** | **Stability** | **Ref.** |
| --- | --- | --- | --- | --- |
| Co_2_P_2_O_7_@NC/CF | 146 mV | 92.6 | 85 h (58%) | [S6] |
| a-CoPSe | 212 mV @100 mA cm^-2^ | 111 | - | [S7] |
| Ni-CoP/Co_2_P | 161 mV | 94 | 15h (80%) | [S8] |
| Co_3_S_4_-MoS_2_ | 260 mV @ 100 mA cm^-2^ | 68.3 | 100 h (57.69%) | [S9] |
| CoO4@MoO_3_ | 125 mV | 94 | 24 h (-) | [S10] |
| Co-Mo-P@C | 162 mV | 34.3 | 20 h (-) | [S11] |
| Co_2_P/CoP@Co@NC | 118 mV | 46 | 50 h (84) | [S12] |
| Fe_0.25_-CoP | 111 | 61.8 | 25 h (96%) | [S13] |
| Co_9_S_8_@MoS_2_/N-HC | 126 | 74.1 | 12 h (89.2) | [S14] |
| CoNiP / CoNi | 160 | 125 | 11 h (-) | [S15] |
| CoNiP/rGO | 209 | 124 | 18 h (-) | [S16] |
| NiCo)_0.85_Se | 169 | 116 | 12 h (-) | [S17] |
| CoP | 160 | 104 | - | [S18] |
| Co_9_S_8_/C | 155 | 200 | - | [S19] |
| CoP-InNC@CNT | 159 | 56 | 20 h (94.0%) | [S20] |
| C @ Co_2_P_2_O_7_/Co_2_P | 137 &  295 @ 100mA cm^-2^ | 85.5 | 47 h (83.15%) | This work |

**References**

[S1] G. Kresse, J. Furthmüller, Efficient iterative scheme for ab initio total-energy calculations using a plane-wave basis set, *Phys. Rev*. B 1996, 54, 11169.

[S2] P. E. Blöchl, Projector augmented-wave method, *Phys. Rev*. B 1994, 50, 17953

[S3] G. Kresse, D. Joubert, From ultrasoft pseudopotentials to the projector augmented-wave method, *Phys. Rev*. B 1999, 59, 1758.

[S4] B. Hammer, L. B. Hansen, J. K. Nørskov, *Phys. Rev*. B 1999, 59, 7413.

[S5] K. Nørskov, T. Bligaard, A. Logadottir, J. R. Kitchin, J. G. Chen, S. Pandelov, J. K. Norskov, *J. Electrochem. Soc*., 2005, 152, J23

[S6] Y. Han, J. Lin, H. Zhou, L. Guo, Y. Wang, Self-standing Co2P2O7@N-doped carbon/carbon foam for hydrogen evolution reaction in alkaline medium, *Diam. Relat. Mater*. 135 (2023) 109843.

[S7] Y. Shi, S. Zhou, J. Liu, X. Zhang, J. Yin, T. Zhan, Y. Yang, G. Li, J. Lai, L. Wang, An integrated amorphous cobalt phosphoselenide electrocatalyst with high mass activity boosts alkaline overall water splitting, *Appl. Catal. Environ*., 341 (2024) 123326.

[S8] P. Arunkumar, S. Gayathri, J.H. Han, Impact of an incompatible atomic nickel-incorporated metal-organic framework on phase evolution and electrocatalytic activity of Ni-doped cobalt phosphide for the hydrogen evolution reaction, *ACS Appl. Energy Mater*. 5 (2022) 2975-2992.

[S9] J. Luo, Y. Zhou, Y. Tuo, Y. Gu, X. Wang, Q. Guo, C. Chen, D. Wang, S. Wang, J. Zhang, Interfacial polarization in ultra-small Co3S4-MoS2 heterostructure for efficient electrocatalytic hydrogen evolution reaction, *Appl. Mater. Today* 26 (2022) 101311.

[S10] I. Ahmed, R. Biswas, R.A. Patil, K.K. Halder, H. Singh, B. Banerjee, B. Kumar, Y.R. Ma, K.K. Haldar, Graphitic carbon nitride composites with MoO3-decorated Co3O4 nanorods as catalysts for oxygen and hydrogen evolution, *ACS Appl. Nano Mater*., 4 (2021) 12672-12681.

[S11] L. Gong, K. Lan, X. Wang, X. Huang, P. Jiang, K. Wang, M. Yang, L. Ma, R. Li, Carbon-coated Co-Mo-P nanosheets supported on carbon cloth as efficient electrocatalyst for hydrogen evolution reaction, *Int. J. Hyd. Energy* 45 (2020) 544-552.

[S12] Z. Lu, Y. Cao, J. Xie, J. Hu, K. Wang, D. Jia, Construction of Co2P/CoP@Co@NCNT rich-interface to sunergistically promote overall water splitting, *Chem. Eng. J*., 430 (2022) 132877.

[S13] Q. Yang, H. Dai, W. Liao, X. Tong, Y. Fu, M. Qian, T. Chen, Construction of Fe-doped CoP with hybrid nanostructures as a bifunctional catalyst for overall water splitting, *Dalton Trans*., 50 (2021) 18069.

[S14] M. Kim, H. Seok, N.C.S. Selvam, J. Cho, G.H. Choi, M.G. Nam, S. Kang, T. Kim, P.J. Yoo, Kirkendall effect induced bifunctional hybrid electrocatalyst Co9S8@MoS2/N-doped hollow carbon) for high performance overall water splitting, *J. Power Sources* 493 (2021) 229688.

[S15] P. Arunkumar, S. Gayathri, J.H. Han, A complementary Co-Ni phosphide/bimetallic alloy-interspersed N-doped graphene electrocatalyst for overall alkaline water splitting, *ChemSuSChem* 14 (2021) 1921-1935.

[S16] J. Li, M. Yan, X. Zhou, Z-Q. Huang, Z. Xia, C-R. Chang, Y. Ma, Y. Qu, Mechanistic insights on ternary Ni_2_-xCoxP for hydrogen evolution and their hybrids with graphene as highly efficient and robust catalysts for overall water splitting, *Adv. Fun. Mater*. 26 (2016) 6785-6796.

[S17] K. Xiao, L. Zhou, M. Shao, M. Wei, Fabrication of (Ni,Co)0.85Se nanosheet arrays derived from layered double hydroxides toward largely enhanced overall water splitting, *J. Mater. Chem*. A 6 (2018) 7585-7591.

[S18] Z. Yin, C. Zhu, C. Li, S. Zhang, X. Zhang, Y. Chen, Hierarchical nickel-cobalt phosphide yolk-shell spheres as highly active and stable bifunctional electrocatalysts for overall water splitting, *Nanoscale* 8 (2016) 19129-19138.

[S19] G. Yilmaz, K.M. Yam, C. Zhang, H.J. Fan, G.W. Ho, In situ transformation of MOFs into layered double hydroxide embedded metal sulfides for improved electrocatalytic and supercapacitive performance, *Adv. Mater*. 29 (2017) 1606814.

[S20] L. Chai, Z. Hu, X. Wang, Y. Xu, L. Zhang, T-T. Li, Y. Hu, J. Qian, S. Huang, Stringing bimetallic metal-organic framework-derived cobalt phosphide composite for high-efficiency overall water splitting, *Adv. Sci*., 7 (2020) 1903195.

**Table S3.** Comparison of HER performance of c-Co-N with recent literatures.

|  | **Anode Electrocatalyst** | **Cathode electrocatalyst** | **Electrolyte** | **Operating Temperature (°C)** | **AEM performance** | **Ref.** |
| --- | --- | --- | --- | --- | --- | --- |
| 1 | CuCoO on Ni (7.4 mg/cm^2^) | Pt/C (1 mg/cm^2^) | 1.0 M KOH | 45°C | 2.25 A/cm^2^ at 1.9V | ^[S21]^ |
| 3 | NiMn_2_O_4_ (3.0 mg/cm^2^) | Pt-C (0.4 mg_pt_/cm^2^) | 1.0 M KOH | 50°C | 0.520 A/cm^2^ at 1.85 V | ^[S22]^ |
| 4 | Ni-FeOOH (4.0 mg/cm^2^) | Pt-C (1.0 mg_pt_/cm^2^) | 1.0 M KOH | 50°C | 0.92 A/cm^2^ at 1.7 V | ^[S23]^ |
| 5 | Ce_0.2_MnFe_1.8_O_4_ (3.5 mg/cm^2^) | Ni (3.5 mg/cm^2^) | 1.0 M KOH | 25°C | 0.3 A/cm^2^ at 1.8 V | ^[S24]^ |
| 7 | CuCoO_x_ (36 mg/cm^2^) | Ni/(CeO_2_-La_2_O_3_)/C (7.4 mg/cm^2^) | 1.0 M KOH | 43°C | 0.47 A/cm^2^ at 1.89 V | ^[S25]^ |
| 8 | NiCo_2_O_4_ (2.0 mg/cm^2^) | Pt–C core-shell@h-MoS_2_/GNF (0.2 mg_pt_/cm^2^) | 1.0 M KOH | 20°C | 20 mA/cm^2^ at 1.64 V | ^[S26]^ |
|  |  |  |  | 60°C | 20 mA/cm^2^ at 1.55 V |  |
| 9 | NiCo_2_O_4_ (2.0 mg/cm^2^) | Pt@Co-NPC-800 (0.2 mg_pt_/cm^2^) | 1.0 M KOH | 20°C | 50 mA/cm^2^ at 1.69 V | ^[S27]^ |
|  |  |  |  | 60°C | 50 mA/cm^2^ at 1.65 V |  |
| 10 | NiCo_2_O_4_ (2.0 mg/cm^2^) | c-Co-N (2 mg/cm^2^) | 1.0 M KOH | 20°C | 186 mA/cm^2^ at 2.2 V | This work |
|  |  |  |  | 60°C | 500 mA /cm2 at 2.2 V |  |

**References**

[S21] Y. S. Park, J. Yang, J. Lee, M. J. Jang, J. Jeong, W. S. Choi, Y. Kim, Y. Yin, M. H. Seo, Z. Chen, S. M. Choi, *Appl. Catal. B Environ.* **2020**, *278*, 119276.

[S22] J. E. Park, H. E. Bae, M. Karuppannan, K. M. Oh, O. J. Kwon, Y. H. Cho, Y. E. Sung, *J. Ind. Eng. Chem.* **2022**, *109*, 453.

[S23] Y. S. Park, J. Lee, M. J. Jang, J. Yang, J. Jeong, J. Park, Y. Kim, M. H. Seo, Z. Chen, S. M. Choi, *J. Mater. Chem. A* **2021**, *9*, 9586.

[S24] T. Pandiarajan, L. J. Berchmans, S. Ravichandran, *RSC Adv.* **2015**, *5*, 34100.

[S25] C. C. Pavel, F. Cecconi, C. Emiliani, S. Santiccioli, A. Scaffidi, S. Catanorchi, M. Comotti, *Angew. Chemie Int. Ed.* **2014**, *53*, 1378.

[S26] S. Ramakrishnan, S. Vijayapradeep, S. C. Selvaraj, J. Huang, S. C. Karthikeyan, R. Gutru, N. Logeshwaran, T. Miyazaki, M. Mamlouk, D. J. Yoo, *Carbon N. Y.* **2024**, *220*, 118816.

[S27] M. R. Subramaniam, S. Ramakrishnan, S. Sidra, S. C. Karthikeyan, S. Vijayapradeep, J. Huang, M. Mamlouk, D. H. Kim, D. J. Yoo, *J. Mater. Chem. A* **2024**, *12*, 5967.

**Table S4.** Comparative table of elements present in c-Co-N before and after cycle stability by the inductively coupled plasma-optical emission spectrometry (ICP-OES)

| Elements | Before Stability | After Stability |
| --- | --- | --- |
| Co | 4.6% | 2.6% |
| P | 1.6% | 0.65% |
| Note : 1% = 10,000 ppm | | |
